# Isolation of Myenteric and Submucosal Plexus from Mouse Gastrointestinal Tract and Subsequent Co-Culture with Small Intestinal Organoids

**DOI:** 10.3390/cells13100815

**Published:** 2024-05-10

**Authors:** Cristina Llorente

**Affiliations:** Department of Medicine, University of California San Diego, La Jolla, CA 92093, USA; allorenteizquierdo@health.ucsd.edu

**Keywords:** enteric nervous system (ENS), three-dimensional (3D), pluripotent stem cells (PSCs), small intestinal organoids, myenteric neurons, submucosal neurons

## Abstract

Intestinal homeostasis results from the proper interplay among epithelial cells, the enteric nervous system (ENS), interstitial cells of Cajal (ICCs), smooth muscle cells, the immune system, and the microbiota. The disruption of this balance underpins the onset of gastrointestinal-related diseases. The scarcity of models replicating the intricate interplay between the ENS and the intestinal epithelium highlights the imperative for developing novel methods. We have pioneered a sophisticated tridimensional in vitro technique, coculturing small intestinal organoids with myenteric and submucosal neurons. Notably, we have made significant advances in (1) refining the isolation technique for culturing the myenteric plexus, (2) enhancing the isolation of the submucosal plexus—both yielding mixed cultures of enteric neurons and glial cells from both plexuses, and (3) subsequently co-culturing myenteric and submucosal neurons with small intestinal organoids. This co-culture system establishes neural innervations with intestinal organoids, allowing for the investigation of regulatory interactions in the context of gastrointestinal diseases. Furthermore, we have developed a method for microinjecting the luminal space of small intestinal organoids with fluorescently labeled compounds. This technique possesses broad applicability such as the assessment of intestinal permeability, transcytosis, and immunocytochemical and immunofluorescence applications. This microinjection method could be extended to alternative experimental setups, incorporating bacterial species, or applying treatments to study ENS-small intestinal epithelium interactions. Therefore, this technique serves as a valuable tool for evaluating the intricate interplay between neuronal and intestinal epithelial cells (IECs) and shows great potential for drug screening, gene editing, the development of novel therapies, the modeling of infectious diseases, and significant advances in regenerative medicine. The co-culture establishment process spans twelve days, making it a powerful asset for comprehensive research in this critical field.

## 1. Introduction

To explore the intricate nuances of intestinal functionality and unravel the molecular mechanism underlying various diseases, three-dimensional (3D) cell culture techniques emerge as indispensable instruments. The advent of organoids has unlocked a territory of possibilities in cultivating fully formed crypt-villus structures that faithfully mimic the intricate workings of intestinal physiology [1]. In vitro models centered on neuronal cultures offer valuable insights into the physiological and pathological functions of the enteric nervous system (ENS). Yet, the paucity of existing models to study the interactions between the ENS and the intestinal epithelium highlights the need for pioneering techniques to be developed.

Here, we introduce an innovative state-of-the-art three-dimensional in vitro technique that adeptly co-cultures small intestinal organoids with both myenteric and submucosal neurons. Through the refinement of isolation methodologies, our approach leads to the creation of mixed cultures incorporating enteric neurons and glial cells from both plexuses. This co-culture system establishes innervations with intestinal organoids, offering a unique opportunity to investigate regulatory interactions. This manuscript and the review manuscript preceding this one published in the same issue, not only highlight significant advances in intestinal organoid and ENS techniques, offering a comprehensive comparison with the presented approach, but also provide an insightful understanding of the pivotal role of both systems in intestinal physiology, disease, and the potential applications of these cutting-edge methodologies, all based on the latest breakthroughs in the field.

### Experimental Design of a Novel Co-Culture Technique

For an in-depth background, we recommend reading the review manuscript preceding this one. This approach will facilitate a comprehensive understanding of the technique and its practical application.

Building upon enteric neuron and organoid culture advances, we have successfully pioneered an innovative 3D in vitro technique that facilitates the ex vivo co-culture of small intestinal organoids in juxtaposition to myenteric and submucosal neurons. The technique involves a careful three-step process of isolating cells. We have made notable progress in the following areas: (1) improving the method used to isolate the myenteric plexus, which now allows us to grow a mixed culture containing both enteric neurons and glial cells; (2) refining the isolation of the submucosal plexus, resulting in a mixed culture of submucosal enteric neurons and glial cells; and (3) co-culturing the myenteric and submucosal neurons with small intestinal organoids that we generate from ISCs after crypt isolation. By co-culturing enteric neurons with small intestinal organoids, we establish neural innervations with various cell types derived from ISC. This innovative co-culture technique holds great promise for studying the complex interactions between enteric neurons and the intestinal epithelium, as well as for investigating the underlying mechanisms of gastrointestinal disorders.

**Figure 1:**
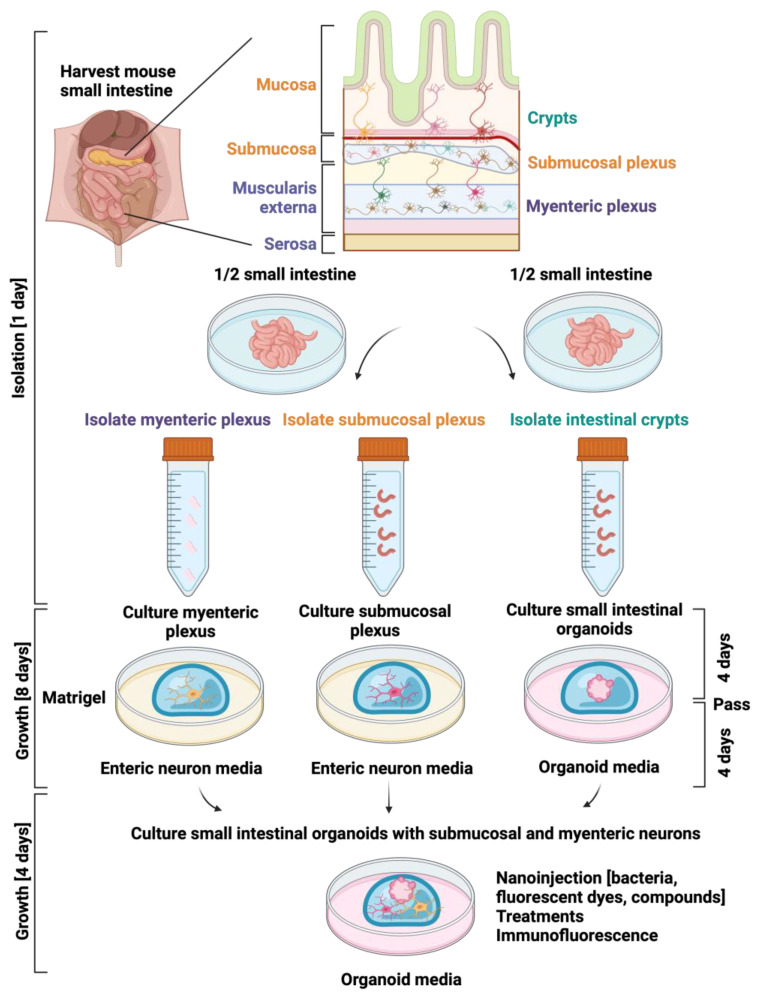
Diagram depicting the stepwise process of the isolation, growth, culture, and the final co-culture of the myenteric plexus, the submucosal plexus, and the small intestinal crypts. (1) Isolation of myenteric and submucosal plexuses: Half of the intestine comprising ¼ of the duodenum and ¼ of the ileum is utilized to isolate the myenteric plexus through mechanical separation and rupture of the muscular externa. The growth and culture of the muscularis externa results in a mixed culture comprising enteric neurons and glial cells derived from the myenteric plexus. Once the muscularis externa is separated, the other layers of this half of the intestine are utilized to isolate the submucosal plexus through enzymatic digestion. Over an eight-day period, within a specialized enteric neural medium, the cultures undergo a progressive growth and expansion of neurites. (2) Isolation of small intestinal crypts: The second half of the intestine is allocated to isolating small intestinal crypts, which are then cultured in an organoid medium for an initial four-day period. Subsequently, they undergo passaging and continue to be cultured for an additional four days. (3) Co-culture system: On the eighth day, when the cultures have reached the appropriate stage, myenteric and submucosal neurons, along with small intestinal organoids, are grown and cultured under specific conditions. This process promotes their survival and proliferation, and in addition facilitates the establishment of innervations with the small intestinal organoids, creating a physiologically relevant model. Figure created with BioRender.com, (accessed on 2 November 2023).

The step-by-step process for isolating, growing, and co-culturing the myenteric plexus, submucosal plexus, and small intestinal crypts involves a systematic approach. Firstly, the myenteric and submucosal plexuses are isolated from one-half of the intestine, with a quarter from the duodenum and a quarter from the ileum, both utilized for isolating both plexuses. The muscularis externa is them employed to cultivate a mixed culture of enteric neurons and glial cells from the myenteric plexus. Concurrently, the submucosal plexus is isolated from the remaining layers through enzymatic digestion, followed by progressive growth and neurite expansion within a specialized enteric neural medium over eight days. The second stage entails isolating small intestinal crypts from the other half of the intestine, with initial organoid culture for four days, followed by passaging and an additional four days of culture. Finally, the co-culture system is established on the eighth day, combining myenteric and submucosal neurons with small intestinal organoids under specific conditions. This synergistic process enhances survival and proliferation, and in addition facilitates the establishment of innervations with the small intestinal organoids, creating a physiologically relevant model for further research (Figure 1).

## 2. Materials and Methods

### 2.1. Reagents and Material Setup

#### 2.1.1. Neuron Isolation Reagents

Krebs Solution (Table 1):

1. Measure out the appropriate amounts of each compound based on the desired final volume.

Caution:

- Sodium chloride (NaCl): Causes serious eye irritation. Avoid contact with eyes.
- Magnesium sulfate (MgSO_4_): Sodium dihydrogen phosphate (NaH_2_PO_4_). The substance has warnings for being harmful through ingestion, skin contact, and inhalation due to acute toxicity.
- Calcium chloride (CaCl_2_): Causes serious eye irritation. Avoid contact with eyes.
- For all: Do not breathe dust. Do not get in eyes, on skin, or on clothing. Wear protective gloves/protective clothing/eye protection/face protection. Wash hands thoroughly after handling.

2. Dissolve the measured amounts of each compound in double-distilled water (ddH_2_O). Dissolve them completely to ensure a homogeneous solution.
3. Adjust the pH of the solution to 7.4 using a pH meter and a suitable pH-adjusting agent such as hydrochloric acid (HCl) or sodium hydroxide (NaOH).

Caution:

- Hydrochloric acid (HCl): The substance is dangerous, causing severe skin burns, eye damage, and acute toxicity when inhaled.
- Sodium hydroxide (NaOH): Causes severe skin burns and eye damage.

4. Once the pH is adjusted, check the osmolality of the solution to ensure it is within the desired range. The osmolality should typically be around 280–320 mOsm/kg.
5. Filter the solution using a sterile filter (pore size of 0.22 μm) to remove any particulate matter or contaminants.
6. Supplement with the correspondent amount of antibiotic/antimycotic solution.
7. Transfer the filtered Krebs solution to a sterilized container or aliquot into smaller volumes for convenient use.

Digestion Solution for Submucosal Plexus (Table 2):

Critical: Gibco™ Collagenase Type II activity is guaranteed to be greater than 125 units/mg.

1. Measure out the appropriate amounts of each compound based on the desired final volume.
2. Dissolve the measured amounts in carbogen-bubbled Krebs solution.
3. Filter the solution using a sterile filter (pore size of 0.22 μm) to remove any particulate matter or contaminants.
4. Place on ice until use.

Reagents to Prepare Enteric Neuron Media:

In the process of preparing the enteric neuron media, it is necessary to prepare an antibiotic cocktail (20X), neurobasal A media containing 0.2X antibiotics, Glial-Derived Neurotrophic Factor (GDNF) solution, and enteric neuron.

- Antibiotic cocktail (20X) (Table 3):

This antibiotic cocktail will be utilized to prepare enteric neuron media and small intestinal crypts media:

Caution:

- Ampicillin sodium: The substance may trigger allergic skin reactions and can also cause allergy or asthma symptoms, leading to breathing difficulties if inhaled.
- Gentamycin: The substance presents various hazards, including the potential for allergic skin reactions, damage to fertility or the unborn child, harm to organs through prolonged exposure, and being very toxic to aquatic life, both acutely and with long-lasting effects.
- Gibco™ Antibiotic/Antimycotic, 100X: Reproductive Harm
- Neomycin: The substance is entirely harmful if swallowed, and it has a full likelihood of causing allergic skin reactions as well as allergy or asthma symptoms when inhaled.
- Metronidazole: The substance carries various hazards, including being harmful if swallowed, in contact with skin, or inhaled, with warnings about acute toxicity. It is also associated with potential genetic defects, carcinogenicity (both suspected and confirmed), and causing damage to organs through prolonged exposure, as well as being hazardous to aquatic life with long-lasting effects.
- Vancomycin hydrochloride: The substance is capable of causing skin irritation, allergic skin reactions, serious eye irritation, and respiratory irritation, prompting warnings for skin, eye, and respiratory effects.

1. Ampicillin solution: Weigh 1 g of sodium ampicillin and dissolve in 10 mL of ddH_2_O to achieve a concentration of 100 mg/mL. Filter the ampicillin solution using a sterile filter with a pore size of 0.22 μm by first prewashing the filter with some ddH_2_O run through it. Aliquot ampicillin solution into 2 mL aliquots and store at −20 °C for a period of one year.
2. Gibco™ Antibiotic/Antimycotic solution, 100X: Prepare 5 mL aliquots and store them at −20 °C. Gibco™ Antibiotic-Antimycotic solution contains 10,000 units/mL of penicillin, 10,000 µg/mL of streptomycin, and 25 µg/mL of Gibco Amphotericin B to avoid bacterial and fungal contamination.
3. Metronidazole solution, 50 mg/mL: Weigh 500 mg of metronidazole and dissolve in 10 mL of ddH_2_O to achieve a concentration of 50 mg/mL. Filter the metronidazole solution using a sterile filter with a pore size of 0.22 μm by first prewashing the filter with some ddH_2_O run through it. Aliquot metronidazole solution into 2 mL aliquots and store at 4 °C for up to two years.
4. Vancomycin hydrochloride solution, 200 mM: First, calculate the mass of vancomycin hydrochloride powder required to prepare the desired concentration: Mass (g) = (Concentration (M) × Volume (L) × Molecular weight (g/mol)). In this case, the concentration is 200 mM (0.2 M), the volume is 500 μL (0.0005 L), and the molecular weight of vancomycin hydrochloride is 1485.71 g/mol. Therefore, mass (mg) = (0.2 M × 0.0005 L × 1485.71 g/mol) × 1000 = 148.571 mg. Weigh 148.5 mg of vancomycin hydrochloride powder and dissolve in 500 μL of ddH_2_O. Mix the contents thoroughly until the powder is completely dissolved. Prepare 170 μL aliquots and store them at −20 °C protected from light for a period of 14 days.

To prepare 10 mL, 20X, add the calculated volumes of antibiotic solutions indicated in the table to 4.44 mL of phosphate-buffered saline (PBS) 1X. Mix thoroughly the cocktail solution and prepare multiple aliquots in volumes of 2 mL each. Use sterile pipette tips and 2 mL sterile microcentrifuge tubes to avoid contamination. It is advisable to label each aliquot with the concentration and date for proper identification. Store at −20 °C.

- Neurobasal A media containing 0.2X antibiotics (Table 4):

1. Add 5 mL of the antibiotic cocktail solution to the 500 mL bottle of Neurobasal A media with a sterile serological pipet under sterile conditions to maintain the media sterile.
2. Mix the contents thoroughly using a 50 mL serological pipet under sterile conditions.
3. Once all the ingredients are added, securely cap the bottle.
4. Label the bottle with the date and contents for proper identification.
5. Store the bottle in a refrigerator at 4 °C until it is used for the preparation of enteric neuron media.

- Glial-Derived Neurotrophic Factor (GDNF) solution (Table 5):

1. Add 1 mL of sterile ddH_2_O to the tube containing 10 μg of GDNF from mouse.
2. Mix the solution: Gently vortex or pipette up and down the contents of the tube to ensure thorough mixing and dissolution of GDNF in the ddH_2_O. This will result in a solution with a concentration of 10 μg/mL.
3. Aliquot the solution: Prepare multiple aliquots of the GDNF solution in volumes of 50 μL each. Use sterile pipette tips and microcentrifuge tubes to avoid contamination.
4. Store at −80 °C

GDNF is frequently used in neuronal culture experiments due to its neuroprotective and neurotrophic properties. It can enhance the survival, growth, and differentiation of neurons, particularly dopaminergic neurons. GDNF also promotes the formation of neurites and axons, contributing to the establishment of neuronal networks [2,3].

- Enteric neuron media (Table 6):

Sodium pyruvate offers an additional source of energy. GlutaMAX is an excellent supplement for neuronal culture as it contains the non-essential amino acid L-glutamine. Neuronal terminals produce glutamate from glutamine. While evidence supporting glutamine’s neurotransmitter function in the central nervous system is limited [4], it is crucial to recognize its metabolic significance [5].

1. Add the calculated volume of supplements and solutions to 45.45 mL of Neurobasal A media and using a serological pipet mix the media under sterile conditions to prepare a final volume of 50 mL.
2. Once all the components are added, securely cap, label, and date the conical tube for proper identification.
3. Store the bottle in a refrigerator at 4 °C until it is used.

Critical:

- Fetal Bovine Serum (FBS) is essential for optimal neuronal growth by providing essential nutrients and promoting cell proliferation.
- Glial-Derived Neurotrophic Factor (GDNF) is vital for both neuronal and glial growth, ensuring the development and maintenance of healthy neurons and supporting the growth of glial cells, which play crucial roles in neural function and support.

#### 2.1.2. Small Intestinal Organoid Culture Materials and Reagents

Small Intestinal Organoid Culture Materials (Table 7):

Small Intestinal Organoid Culture Reagents (Table 8):

Caution:

- N-Acetylcysteine: The substance may cause skin and respiratory irritation, and it is highly likely to cause serious eye irritation, warranting warnings for skin, eye, and respiratory effects.
- Ethylenediaminetetraacetic acid (EDTA): H319 indicates a warning for serious eye irritation or damage.
- HA-R-Spondin1-Fc 293T Cells: Caution should be exercised, and the cell line should be handled as potentially bio-hazardous material using Biosafety Level (BSL)-2 containment. Appropriate safety procedures should be used when handling all cell lines. Regular monitoring for mycoplasma contamination is essential to ensure the safety and integrity of cell culture experiments.
- Follow additional steps to prepare crypt culture reagents:
- Preparation of 4 mL solution of 0.1% BSA in PBS 1X (Table 9):

1. First, prepare a 4 mL solution of 0.1% BSA in PBS 1X.
2. Weigh 4 mg of BSA and add a small volume of PBS 1X. Ensure that the BSA is fully dissolved.
3. Add enough PBS 1X to reach a final volume of 4 mL. Mix the solution gently to ensure it is homogeneous.
4. Filter the solution (pore size of 0.22 μm) and maintain at 4 °C.

- Preparation of 100 μg/mL solution of mNoggin in 0.1% BSA in PBS 1X to create a 1000X stock solution (Table 10):

5. Take 1 mL of the prepared 0.1% BSA in PBS solution and add it to 100 μg of mNoggin (PreproTech, 250-38).
6. Mix thoroughly to ensure proper mixing of the components.
7. Finally, aliquot the solution into 20 μL portions.
8. Store them at −80 °C for future use.

mNoggin acts as an inhibitor of bone morphogenetic proteins (BMPs), which are signaling molecules involved in the regulation of cell differentiation and tissue development. By inhibiting BMP signaling, mNoggin helps to maintain the self-renewal and pluripotency of intestinal stem cells within the crypts. During the isolation of crypts, mNoggin is used to create an environment that supports the survival and growth of the stem cells. It is often added to the culture medium to prevent the differentiation of stem cells into other cell types and to maintain their undifferentiated state [6].

- Preparation of 500 μg/mL murine Epidermal Growth Factor (mEGF) Recombinant Protein in 0.1% BSA in PBS 1X to create a 10,000× stock solution (Table 11).

1. Take 2 mL of the prepared 0.1% BSA in PBS 1X solution and add it to 1 mg of Gibco™ mEGF (Fisher Scientific, MG8043).
2. Mix thoroughly to ensure proper mixing of the components.
3. Prepare 5 μL aliquots of the solution.
4. Store the aliquots at −80 °C for future use.

mEGF is essential for small intestinal organoid growth as it activates the RAS/RAF/MEK/ERK signaling pathway [7], stimulates the migration and proliferation of intestinal stem cells, and inhibits apoptosis [8,9]. These effects promote the expansion and maintenance of small intestinal organoids, ensuring their development into functional three-dimensional structures that closely mimic the characteristics of the small intestine.

- Preparation of 500 mM N-Acetylcysteine stock solution (Table 12):

1. Calculate the mass of N-Acetylcysteine powder required to prepare 5 mL at a concentration of 500 mM: Mass (mg) = (0.5 M × 0.005 L × 163.19 g/mol) × 1000 = 407.975 mg.
2. Weigh 408 mg of N-Acetylcysteine powder (Millipore Sigma, A9165-5G), and dissolve in 5 mL of sterile ddH_2_O.
3. Mix thoroughly until the powder is completely dissolved.
4. Prepare 200 μL aliquot.
5. Store at −20 °C protected from light.

Caution:

- N-Acetylcysteine: The substance may cause skin and respiratory irritation, and it is highly likely to cause serious eye irritation, warranting warnings for skin, eye, and respiratory effects.
- Preparation of R-Spondin 1 conditioned media:

1. R-Spondin 1 secreted protein is expressed by stable transfected 293T cells. These cells are selected using zeocin and express mouse R-spondin 1 protein tagged with hemagglutinin (HA) at the C-terminus and Fc at the N-terminus in the conditioned medium. This cell line was generously provided by the Calvin Kuo Lab at Stanford University and it is now distributed by R&D Systems (Catalog # 3710-001-01). Please refer to R&D Systems (Catalog # 3710-001-01) for protocols to culture, pass, freeze, and collect HA-R-Spondin 1-Fc conditioned media.
2. Prepare 1 mL aliquots of the collected conditioned media.
3. Store the aliquots at −20 °C for future use.

R-Spondin 1, also known as Cristin 3, is a secreted protein that enhances the canonical Wnt/β-catenin signaling pathway. It achieves this by competing with the Wnt antagonist Dkk-1 and binding to Frizzled-8, Kremen, LRP-6, Lgr4, Lgr5, and Lgr6. Furthermore, R-Spondin 1 enhances the availability of Wnt receptors on the cell surface, thereby promoting crypt proliferation [10].

- Preparation of supplemented advanced DMEM/F12 media (Table 13):

1. Add 5 mL of the GlutaMAX, HEPES, and Antibiotic/Antimycotic solution to a bottle of 500 mL Advanced DMEM/F12.
2. Store at 4 °C.

Once all the components to prepare the small intestinal organoids media have been assembled, proceed to the preparation of small intestinal organoid media:

Small Intestinal Organoid Media (Table 14):

1. Add the calculated volume of supplements and solutions to a 16,328 mL supplemented advanced DMEM/F12 media under sterile conditions to prepare a final volume of 20 mL.
2. Mix thoroughly, securely cap the conical tube, and label appropriately.
3. Store at 4 °C until use.

Critical: Prepare the appropriate quantity based on the scale of the experiment.

Proceed with the preparation of other reagents necessary for crypt isolation:

- Preparation of EDTA 2 mM in Dulbecco’s phosphate-buffered saline (DPBS) 1X solution (Table 15):

4. Add 200 μL of the 0.5 M EDTA to the 49.8 mL of DPBS 1X.
5. Place the solution on ice to keep it cold.

Caution: Ethylenediaminetetraacetic acid (EDTA): H319 indicates a warning for serious eye irritation or damage.

- Prepare DPBS 1X solution with 10% FBS (Table 16):

1. Add 5 mL of the FBS to the 45 mL of DPBS 1X containing antibiotics and antimycotics to create the DPBS 1X/FBS solution for washing steps.
2. Place the mixture on ice to keep it cold.

Once the materials and reagents are ready, proceed with the indicated steps to successfully co-culture myenteric and submucosal neurons. This process involves several steps: First, the small intestine is harvested, and then the myenteric and submucosal plexuses are isolated from half of the intestine, resulting in a mixed culture of enteric neurons and glial cells. Simultaneously, small intestinal crypts are isolated from the other half of the intestine and grown into organoids. After eight days, both cultures are at an appropriate stage for co-culturing. The co-culture approach promotes the survival, growth, and development of innervations between myenteric and submucosal neurons and small intestinal organoids (Figure 1).

### 2.2. Procedure

#### 2.2.1. Harvesting of Mouse Small Intestine

1. C57BL/6J female or male mice (age, 8–12 weeks old) were used for the experiments.
2. Euthanize the mouse following institutional ethics committee protocols.

Caution: All studies should be approved by applicable national and institutional animal care and use committees.

**Figure 2:**
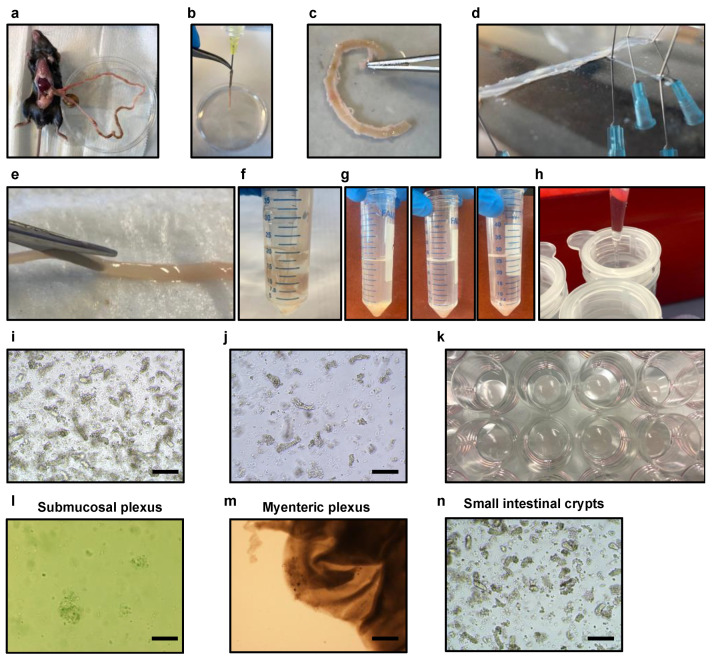
Critical steps in the process of isolation of myenteric plexus, submucosal plexus, and crypts. (**a**) The small intestine is harvested from a mouse, initiating the isolation procedure. (**b**) A photograph captures the process of flushing the intestinal contents with DPBS 1X. (**c**) The mesenteric tissues, blood vessels, and fat are removed from the surface of the intestine. (**d**) Picture depicting the harvesting of the mucularis externa with serosa along the length of the intestine used for isolation of the myenteric plexus. (**e**) Before small intestinal crypts isolation, the intestine is longitudinally cut using surgical scissors, exposing the lumen of the intestine. (**f**) The small intestine is cut into small fragments measuring 2–4 mm and transferred to a 50 mL tube containing DPBS 1X. (**g**) Mechanical cleaning of the intestinal debris involves pipetting up and down the fragments using a 50 mL serological pipette, followed by the removal of the dirty supernatant until a clear supernatant is achieved. (**h**) After EDTA dissociation, crypt fractions are collected by filtration using a 70 μm strainer. (**i**) Representative bright-field microscopy image representing the first crypt fraction eluted, displaying multiple crypts along with other epithelial cells. Scale bars = 200 μm. (**j**) Representative bright-field microscopy image of the fourth crypt fraction eluted characterized by cleaner and purer crypts (reduced presence of epithelial cells). Scale bars = 200 μm. (**k**) Isolated myenteric plexus, submucosal plexus, and crypts are plated inside Matrigel drops of 30 μL in a pre-warmed 24-well plate. (**l**) Representative bright-field microscopy image depicting the isolated submucosal plexus on the first day after isolation. The isolated submucosal components are not readily discernible. Scale bars = 50 μm. (**m**) Representative bright-field microscopy image depicting the plated muscularis externa containing the myenteric plexus on the first day after isolation. The neuronal components are not readily discernible. Scale bars = 400 μm. (**n**) Representative bright-field microscopy image depicting isolated crypts on the first day after isolation. Scale bars = 400 μm.

3. Place Krebs solution on ice and bubble it with carbogen (95% oxygen, 5% CO_2_) for at least 30 min to stabilize pH before tissue harvesting.
4. Use a single small intestine for the isolation of submucosal and myenteric enteric neurons together with glia cell and small intestinal crypts.
5. Prepare the surgical area (abdominal cavity) with ethanol 70%. Perform a small incision with surgical scissors in the epidermis to expose the dermis of the abdominal cavity. Perform another two incisions in the dermis of the abdominal cavity with a clean surgical scissor to expose the small intestine. Harvest the small intestine from the stomach to the cecum of the mice with forceps and clean surgical scissors and place it in a 100 mm cell culture dish containing 10 mL of cold and sterile DPBS 1X supplemented with antibiotics and antimycotics (Figure 2a).

6. Gently flush small intestinal contents with a 30 mL syringe with a needle (20G) containing sterile and cold DPBS 1X from proximal to distal in a 100 mm cell culture dish until clean (Figure 2b).
7. Remove any mesenteric tissue, blood vessels, and fat from the exterior of the intestine in a clean 100 mm cell culture dish containing sterile DPBS 1X supplemented with antibiotics and antimycotics (Figure 2c).
8. Remove and discard the duodenal bulb and the first 2 cm of the duodenum to avoid the Brunner’s glands.
9. In order to enhance the purity of the isolation procedure, excise the Peyer’s patches along the length of the small intestine.

Critical: Harvesting of the intestine should be performed considering the distinct neuronal cytoarchitecture along the intestine [11]. Therefore, ¼ of the intestine from the duodenal part will be used to isolated both plexuses and another ¼ of the intestine from the ileal part will be used to harvest both plexuses, while the rest ½ of the intestine will be used to isolate intestinal crypts. This will allow us to have higher representative neuronal subtypes in the co-culture.

10. Divide the small intestine into three parts.
11. Place the small intestine designated for the isolation of the myenteric and submucosal plexus into a 100 mm cell culture dish covered with 15 mL of ice-cold Krebs solution.
12. Place the small intestine designated for the isolation of crypts into a 100 mm cell culture dish covered with 15 mL of ice-cold sterile DPBS 1X supplemented with antibiotics and antimycotics.

The first step in our co-culture technique involves the isolation of enteric neurons from the myenteric and submucosal plexus. This is achieved through a combination of enzymatic digestion (submucosal plexus) and mechanical dissociation (myenteric plexus) methods. The harvested neurons are then prepared for co-culture. Simultaneously, small intestinal organoids are generated from isolated crypts using established protocols [12]. Coculturing of the submucosal and myenteric plexuses with small intestinal organoids would allow us to understand the distinct functions of the ENS and its role in regulating the intestinal mucosa.

#### 2.2.2. Isolation of Neurons and Glial Cells from the Myenteric Plexus

1. Take the small intestine designated for the isolation of the plexuses and place it into a 9 cm silicone-coated black petri dish containing 20–30 mL of ice-chill Kreb’s solution supplemented with 1× antibiotic/antimycotics solution (Figure 2d).
2. With the use of surgical forceps and a 27G ½ inch needle, secure the proximal and distal parts of the intestine to the silicone (Figure 2d).
3. Generate a localized region of the myenteric plexus attached to the longitudinal muscle (muscularis externa and serosa) in proximity to the needles by delicately rubbing the outermost layer of the small intestine using a sterile cotton-tipped applicator 6″ 2′s.
4. Using forceps and a pair of scissors, incise the small intestine along the mesenteric line, carefully exposing the lumen.
5. Subsequently, remove the needles and turn the small intestine downwards using a pair of angled forceps, ensuring that the lumen side faces down (Figure 2d).
6. Rearrange the needles to extend the small intestine.
7. Localize the previously excited muscularis externa with serosa and hold it with a pair of angled forceps.
8. Using a wet cotton-tipped applicator, gently pull and recover the complete layer of the muscularis externa with serosa along the entire length of the small intestine. Ensure that the forceps are holding the submucosal layer of the intestine down near the site of separation (Figure 2d).

Critical: Precise handling of the muscularis externa with serosa is crucial for the successful isolation of the myenteric plexus.

9. Take a 15 mL conical tube and add 2 mL of ice-cold Krebs solution.
10. Transfer the isolated muscularis externa with serosa layer to a 100 mm cell culture dish containing an additional 3 mL of Krebs solution.
11. Using a sterilized razor blade, cut the isolated muscularis externa with serosa layer into the smallest possible pieces.

Critical: Sectioning the muscularis externa with serosa, instead of digestion, is essential for the effective isolation of the myenteric plexus.

12. Using a previously cut and sterile 1 mL pipette tip, collect the resulting 3 mL solution containing the minced muscular externa, and transfer it into the 15 mL conical tube already containing the 2 mL of Krebs solution.
13. Place it on ice until further use.

#### 2.2.3. Isolation of Neurons and Glial Cells from the Submucosal Plexus

1. Recover the remaining part of the intestine containing the submucosa and mucosal layer of the intestine by unpinning it from the 9 cm silicone-coated black petri dish.
2. Rinse it in Krebs solution.
3. Place the segments into the digestion solution for the submucosal plexus while cutting them into approximately 2 mm pieces with scissors.
4. Digest it with the prepared digestion solution for the submucosal plexus for 60 min at 37 °C in a water bath with gentle shaking.

Critical: Excessive digestion leads to damage of submucosal neurons.

5. Collect the cells by centrifugation for 8 min at 356× *g* in a refrigerated centrifuge set to 4 °C.
6. Prepare 0.05% trypsin solution by combining 1 mL of 0.25% trypsin and 4 mL of DPBS 1X supplemented with antibiotics and antimycotics in a sterile 50 mL cell culture tube in the cell culture hood.
7. Warm the 0.05% trypsin solution at 37 °C in a water bath.
8. After centrifugation, discard the supernatant without using a vacuum, by decantation into another falcon tube.
9. Carefully add to the cell pellet 5 mL of 0.05% trypsin solution and mix it gently by pipetting up and down.

Critical: It is advisable to monitor for signs of excessive digestion, such as cell clumps, changes in morphology, and decreased viability. This is crucial to prevent potential harm to submucosal neurons. Adjustments to concentration, digestion time, or other factors should be based on careful observation.

10. Incubate the cells for digestion in a 37 °C water bath for 7 min.
11. Neutralize the trypsin by adding 500 μL of cold FBS.
12. Centrifuge the cells for 8 min at 356× *g*.
13. While the cells are being centrifuged, place a sterilized 70 μm filter on top of a sterile 15 mL falcon tube.
14. Remove and discard the supernatant by decantation into another falcon tube.
15. Gently resuspend the cell mixture by gently pipetting in 5 mL of Krebs solution. Do not generate air bubbles.
16. Count the cell number using standard Trypan Blue staining and hemocytometry.
17. Maintain the tube on ice with continuous gentle rotation agitation until subsequent use.

#### 2.2.4. Isolation of Small Intestinal Crypts

Retrieve the designated small intestine intended for the isolation of crypts, which has been previously reserved. Place the intestine in a 100 mm cell culture dish containing 15 mL of ice-cold DPBS 1X supplemented with antibiotics and antimycotics. Proceed by making a longitudinal incision along the entire length of the intestine, creating a flat surface that exposes the lumen of the intestine (Figure 2e).

1. Utilizing surgical scissors, cut the intestine into small 2–4 mm fragments. Transfer these fragments into a 50 mL tube containing 20 mL of ice-cold DPBS 1X supplemented with antibiotics and antimycotics (Figure 2f).
2. Gently and thoroughly cleanse the intestinal fragments by repeatedly pipetting the DPBS 1X solution up and down using a 50 mL serological pipette. Take care to avoid bubble formation during the cleaning process. Allow the intestinal fragments to settle down by gravity, and carefully remove the supernatant containing any debris or impurities. Add fresh ice-cold DPBS 1X supplemented with antibiotics and antimycotics to the tube. Repeat the cleaning process multiple times until the supernatant becomes nearly clear. This requires performing approximately 10 successive washes (Figure 2g).
3. Remove the supernatant and leave the intestinal fragments in the conical tube.
4. In this step, 25 mL of a DPBS 1X solution supplemented with 2 mM of EDTA is added to the tube containing the intestinal fragments. EDTA acts as a dissociation agent by chelating divalent cations, such as calcium and magnesium, which are vital for cell-cell adhesion. By disrupting these interactions, EDTA helps loosen the cell-to-cell attachments, facilitating the isolation of single cells from the intestinal epithelium and the isolation of the crypts.
5. Place the tube on a rocking tube platform within a cold room and allow it to shake for a duration of 30 min to further aid in the dissociation process.

Critical: This step is critical and proper rocking must be attained to ensure detachment of intestinal epithelium.

6. After the incubation period, allow the smaller pieces, primarily consisting of isolated crypts, to undergo gravitational settling.
7. Once settled, the turbulent supernatant, which contains the single cells and dissociated single cells from the intestinal epithelium, can be carefully removed. This step ensures the separation of the desired crypts from the remaining single cells from the epithelium.
8. Rapidly add 25 mL of DPBS 1X solution with 10% FBS to the pellet containing the isolated intestinal crypts to provide nutrients and support for the isolated crypt structures. To extract the first fraction of intestinal crypts, the solution is repeatedly pipetted up and down using a 50 mL serological pipette a least 10 times. This process helps release the crypts from the tissue and promotes their collection.
9. In order to enhance the isolation of crypts and eliminate any residual debris or large clusters, the initial fraction containing the crypts is subjected to filtration utilizing a 70 μm strainer. The tube containing the filtrate is then securely capped and appropriately labeled as the first fraction (Figure 2h).
10. The isolated crypts are transferred back from the 70 μm strainer into a 50 mL tube, and 25 mL of DPBS 1X solution supplemented with 10% FBS is added. The second fraction of crypts is extracted by gently pipetting the solution up and down using a 50 mL serological pipette for 10 cycles. This process helps release additional crypt structures from the tissue fragments.

Critical: This step is critical and proper release of crypts is crucial.

11. The extraction and collection of crypts is repeated for a total of four rounds. After each round, the eluted crypts from the respective fraction are carefully collected and separated. It is important to label each of the four fractions to differentiate and track the isolated crypt populations.

Critical: Avoid mixing fractions; select the one with at least 100 crypts/mL, containing minimal epithelial cells, minimal debris, and the best complete crypt structures.

12. Centrifuge the crypts at 300× *g* to promote sedimentation.
13. Resuspend the pellet in 10 mL ice-cold supplemented advanced DMEM/F12 media and proceed with centrifugation at 168× *g* to remove immune cells. This step helps to enrich the population of intestinal epithelial cells (IECs) by eliminating non-epithelial immune cells.
14. Resuspend the pellets in 10 mL ice-cold supplemented advanced DMEM/F12 media. This step provides a suitable culture medium to maintain the viability and functionality of the crypts.
15. Using a pre-wetted pipette tip, take 10 μL of sample from the selected fraction and place it on a glass slide or hemocytometer for precise crypt counting and detailed morphological examination. The purpose of this step is to assess the characteristics and quantity of the crypts.
16. Utilizing an inverted microscope, carefully examine the size and purity of the crypts, ensuring to avoid fractions that contain a significant number of single cells (Figure 2i,j). Aim to select the fraction or fractions that display a high degree of pure crypts, without substantial contamination by single cells.
17. Estimate the number of crypts per fraction by performing a specific count that focuses solely on the crypts and excludes single cells. This quantification enables the determination of the crypt population within each fraction accurately.
18. To create an optimal three-dimensional growth environment for the crypts, calculate and plate a variable range of 100 to 500 crypts, which should be diluted in 30 μL of liquid ice-cold Matrigel.
19. Carefully place 100–500 crypts, suspended in 30 μL of ice-cold Matrigel, onto a single well of a prewarmed 24-well plate.

Critical: The success of the isolation depends on the precise plating of an appropriate number of crypts. Matrigel undergoes solidification at room temperature (15–25 °C), emphasizing the necessity of rapid work and conducting procedures on ice to maintain its proper handling consistency. It is of utmost importance to thoroughly mix the crypts with the Matrigel solution, taking care to prevent the formation of bubbles. This meticulous mixing process ensures a uniform distribution of the crypts within the Matrigel, promoting their proper embedding and subsequent growth. By providing this three-dimensional environment, the natural architecture of the crypts and their cellular interactions are supported, thereby facilitating their development and maintaining their physiological behavior.

#### 2.2.5. Plating the Myenteric Plexus, the Submucosal Plexus, and Isolated Crypts

Only one-fifth of a halved small intestine will be employed for the plating of the crypts, and the submucosal plexus with all the isolated myenteric plexus will be used for plating.

Critical: Plating submucosal neurons or crypts at a high density will result in failure to form and develop.

1. Thaw aliquots of Matrigel on ice until isolation.
2. Pre-incubate a 24-well plate in a CO_2_-incubator.
3. Plate the dissociated cells into Matrigel, at a density of 1 × 10^5^ enteric neuronal cells derived from the submucosal plexus per 30 μL of Matrigel, which corresponds to one-fifth of a halved small intestine. Therefore, transfer 1 mL of the isolated submucosal plexus (one-fifth) and carefully transfer it to a 15 mL Falcon tube.
4. For the isolation of crypts, collect the necessary volume to obtain a total of 2600–13,000 crypts, which is equivalent to one-fifth of a halved small intestine. Transfer the collected volume to a 15 mL Falcon tube, ensuring the accurate quantification of the required crypts.
5. Centrifuge the isolated submucosal, myenteric enteric neurons, and crypts at 356× *g* for 8 min.
6. Aspirate and discard all the supernatant using a pipette.
7. Resuspend the isolated myenteric plexus in 400 μL of ice-cold liquid Matrigel. This step provides a supportive matrix for the myenteric plexus, promoting its preservation and subsequent growth.
8. Similarly, resuspend the isolated submucosal plexus in 400 μL of ice-cold liquid Matrigel, creating an appropriate environment for the submucosal component.
9. For the isolated crypts, resuspend them in 780 μL of ice-cold liquid Matrigel, facilitating their embedding within the supportive matrix.
10. Plate 12 wells of a pre-warmed 24-well plate with 30 μL of Matrigel containing the isolated components of the submucosal plexus (Figure 2k,l and Supplementary Video SA1).
11. Plate 12 wells of a pre-warmed 24-well plate with 30 μL of Matrigel applied to the center of the well containing the myenteric plexus (Figure 2m).
12. Plate 24 wells of a pre-warmed 24-well plate with 30 μL of Matrigel containing the isolated crypts (Figure 2n and Figure S2).

Critical: Allow the Matrigel to solidify by polymerization for 30 min in the CO_2_ cell incubator, enabling the formation of a stable matrix that supports cellular growth.

13. Invert the plates for an additional 30 min in the CO_2_ cell incubator to promote three-dimensional growth. This inverted position promotes three-dimensional growth, ensuring the crypts, submucosal plexus, and myenteric plexus are appropriately positioned within the Matrigel for their respective growth and development.
14. Add 500 μL per well of warmed enteric neuron media to the isolated submucosal and myenteric plexuses, providing the necessary nutrient-rich medium for their specific requirements.
15. Add 500 μL per well of warmed intestinal organoid media to the isolated small intestinal crypts, ensuring the provision of an appropriate medium to support their growth and maintain their cellular characteristics.

Critical: Cell and tissue cultures are incubated at 37 °C with 5% CO_2_ under normal oxygen levels.

### 2.3. Culture and Expansion of Isolated Enteric Neurons from the Submucosal Plexus, Myenteric Plexus, and Intestinal Crypts

It is important to monitor the morphological changes of the cultured cells daily under the microscope. Upon the isolation of the myenteric plexus and submucosal plexus, the presence of neurons and other cell types may not be readily discernible (Figure 2l,m).

The primary purpose of the enteric neuronal cell culture medium is to support survival of long-term cultures of post-natal and adult primary neurons and neurite outgrowth. Neuronal cultures primarily originate from fully mature neurons, which possess limited or no replicative capacity. However, rodents and humans have ENS neural/progenitor cells in the postnatal period [13], and neural progenitor cells or neural stem cells within the plexuses responsible for self-renewal and differentiation into various types of neuronal and glial cells are also isolated. Therefore, the growth of neurons in culture primarily depends on the survival and maintenance of existing cells and the presence of neural progenitor cells [14,15].

**Figure 3:**
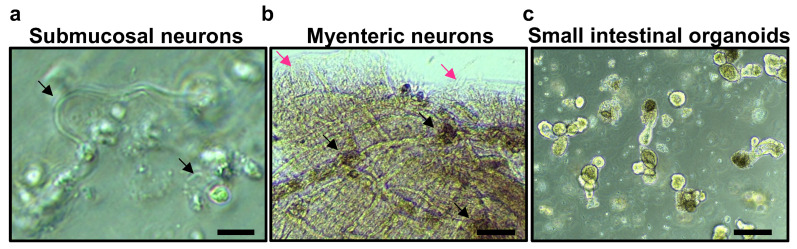
Culture of submucosal, myenteric, and small intestinal organoids four days after plating. (**a**) Representative bright-field microscopy image showing submucosal cellular components (black arrows) grown tridimensionally after four days of culture in neuronal medium. Scale bars = 12.5 μm. (**b**) Representative bright-field microscopy image depicting the myenteric plexus after four days of culture in neuronal medium. Myenteric neurons have the ability to organize and expand, forming ganglion-like plexus structures (black arrows) and project outside the tissue, creating a distinct brush border (magenta arrows). Scale bars = 25 μm. (**c**) Small intestinal organoids after four days of culture in organoid media. Scale bars = 200 μm.

Upon digestion of the submucosal plexus tissues, careful observation reveals the presence of round living cells alongside remnants of tissue debris (Figure 2l and Figure S1). After one day in culture, enteric neurons initiate the growth of their projections known as neurites. However, the precise identification of these neurons may still be somewhat unclear during this initial phase. As the culture progresses, the neurons develop three-dimensionally within the Matrigel substrate. Approximately four days into the culture, distinct morphological features of the neurons become discernible. The cells aggregate around individual ganglia, exhibiting noticeable neurite outgrowth (Figure 3a and Figure S3). These cell clusters are interconnected by extensions of the neurites, resembling the natural arrangement of the submucosal plexus.

Following the isolation of the myenteric plexus, a notable proliferation of new cells becomes evident after a period of four days. As the cells are cultured for four days, some of the neurons exhibit a remarkable tendency to organize themselves into well-defined ganglion-like plexuses within the isolated tissue. Additionally, neurites extend outward from the tissue, generating a brush-like border formation (Figure 3b, magenta arrows; Supplementary Figure S4a–c).

The primary focus of this manuscript revolves around the interconnection of neurites between different neuronal cell types and small intestinal organoids. Further studies are needed to evaluate the functionality of these connections through electrophysiology assays and the assessment of neurotransmitter content in neurites. However, it is important to note that the isolation protocol described herein is not limited to enteric neurons exclusively from the submucosal and mucosal plexus. It also facilitates the isolation of glial cells from various plexuses, as well as smooth muscle cells, ICCs, and fibroblasts. These different cell types can be distinguished through the use of immunofluorescence analyses. Specifically, enteric glia cells can be visualized using an S100 protein, beta polypeptide, neural (S100β) antibody. Enteric smooth muscle cells, on the other hand, can be identified by employing alpha-smooth muscle actin (alpha-SMA) antibodies. Additionally, the presence of fibroblasts can be detected using an anti-Vimentin antibody. By utilizing these immunofluorescence techniques, a more comprehensive understanding of the roles played by different cell types in the regulation of the intestinal mucosa can be assessed. This method would allow for a detailed investigation into the roles played by different cell populations in maintaining the integrity and proper functioning of the intestinal mucosa.

Isolated crypts (Figure 2n) contain stem cells or progenitor cells with the ability to proliferate and differentiate into various cell types facilitated by the intestinal organoid media. This inherent replicative capacity in organoids can contribute to their higher cell growth ratios compared to neuronal cultures. After 4 days, the small intestinal organoids grew adopting the stereotypic budding appearance (Figure 3c) that has been previously described. Due to the distinct growth characteristics of isolated neuronal cultures and small intestinal crypts, it is necessary to culture them separately. Small intestinal crypts, specifically intestinal organoids, exhibit a high proliferative capacity, requiring frequent passaging every four days to maintain their morphology. This passaging step allows ample time for the neuronal cultures to reach an appropriate state for co-culture.

### 2.4. Optimal Passaging of Small Intestinal Organoids and Medium Change for Enteric Neuronal Cell Cultures after Four Days of Initial Culture

Following the initial four days of culture, the enteric neuronal cell culture medium is refreshed to provide optimal conditions for neuronal growth and maturation.

Simultaneously, the small intestinal crypts are passaged to ensure their continued expansion and maintenance of their specific cellular composition and morphology.

Before performing the transfer of the small intestinal crypts, thaw Matrigel on ice, and prepare the intestinal organoid medium. Furthermore, a 24-well plate is pre-warmed in an incubator to create a suitable environment for Matrigel polymerization.

The following steps outline the process of passing the small intestinal organoids:

1. Remove the current culture medium from each well of the 24-well plate using a pipette.
2. Add 500 μL of cold-supplemented advanced DMEM/F12 media to each well and use a pipette with a bent tip to gently break up the gel and the crypts. Transfer the mixture to a 15 mL tube.

Critical: Working under chilled conditions is essential for effectively liquefying the Matrigel and successfully passing the organoids.

3. Spin the tube containing the crypts at 600 rpm at 4 °C.

Critical: Thoroughly remove the old Matrigel, while closely monitoring the small intestinal crypts at the bottom of the conical tube.

4. Discard the supernatant.
5. Add 780 μL of ice-cold liquid Matrigel into the tube, which is equivalent to 30 μL per well. To further fragment the intestinal organoids, employ a pre-wetted pipette with a bent tip, and perform pipetting up and down motions.
6. Position droplets containing 30 μL of crypts embedded in Matrigel at the center of each pre-warmed well in the 24-well plate.
7. Add 500 μL of complete small intestinal growth media to facilitate proper small intestinal growth.
8. Transfer the plate to a CO_2_ incubator at 37 °C.
9. Allow the cells to grow for an additional four days.

### 2.5. Staining Procedures

Tissue samples were fixed in 10% buffered formalin, embedded in paraffin (Paraplast plus, McCornick) and sectioned at 5 μm thickness, and stained with Alexa Fluor^®^ 594 anti-Tubulin β 3 (TUBB3) antibody (1:200) (Biolegend, San Diego, CA, USA, 801208)) primary antibody and biotinylated Ulex Europaeus Agglutinin I (UEA-I) (1:200) (Vector, Pittsburgh, PA, USA, B-1065-2) overnight and then incubated with Streptavidin CY5 (SA-1500-1) secondary antibodies (Vector). Nuclei were stained in blue (represented as gray) with a VectashieldR (Vector Laboratories) mounting medium containing DAPI and imaged by fluorescent microscopy. Control sections were stained with isotype antibody and showed no staining.

For small intestinal organoid staining, media was removed and organoids were fixed in 4% paraformaldehyde (PFA) in PBS 1X at 4 °C for 30 min, and then rinsed in PBS 1X. Samples were immersed in permeabilization buffer (Thermofisher scientific, 00-5523-00) for 30 min followed by a wash in PBS 1X for 5 min at three times each, incubated with two drops of protein block (Agilent, Santa Clara, CA, USA, X090930-2) for 30 min, and stained with anti-MUC2 antibody (Thermofisher scientific, PA5-21329) or Alexa Fluor^®^ 594 anti-Tubulin β 3 (TUBB3) antibody (1:200) (Biolegend, San Diego, CA, USA, 801208) or biotinylated Ulex Europaeus Agglutinin I (UEA-I) (1:200) (Vector, B-1065-2) primary antibodies overnight, and then incubated with donkey anti-rabbit IgG (H + L) highly cross-adsorbed secondary antibody, Alexa Fluor™ 488 (Thermofisher scientific, A21206), or Streptavidin CY5 (SA-1500-1) secondary antibodies (Vector). Nuclei were stained in blue (represented as gray) with a VectashieldR (Vector Laboratories) mounting medium containing DAPI and imaged by fluorescent microscopy. Control sections were stained with isotype antibody and showed no staining. For small intestinal organoid microinjections, 2.5 mg/mL tetramethylrhodamine (TMR)-dextran 10,000 MW, lysine fixable (Life Technologies, Carlsbad, CA, USA, D1817), or 1 mg/mL ovalbumin fluorescein conjugate (Life Technologies, O23020) was used. Next, tissue fixation, permeabilization, blocking, and staining were performed as described above.

## 3. Results

### 3.1. Co-Culture of Small Intestinal Organoids and Isolated Enteric Neurons from Submucosal and Myenteric Plexus

**Figure 4:**
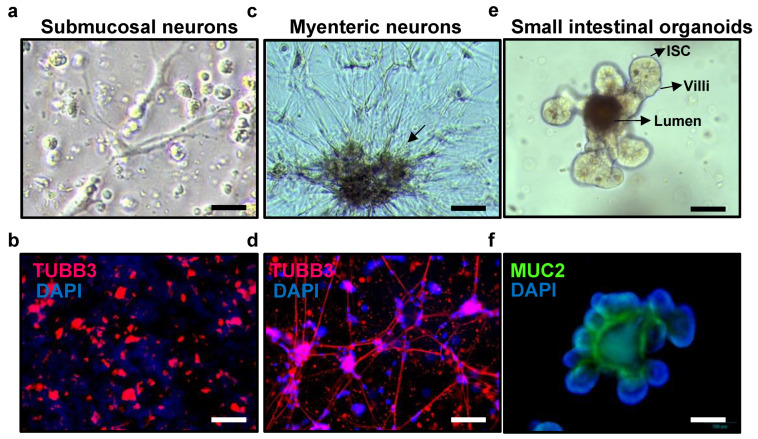
Culture of submucosal neurons, myenteric neurons, and organoids for a duration of eight days, allowing for their growth and development. (**a**) Tridimensional growth of submucosal plexus cellular components after eight days of culture in neuronal medium (bright-field). Scale bars = 12.5 μm. (**b**) Representative immunofluorescent image of submucosal neurons after eight days of tridimensional culture in neuronal medium probed tubulin beta-III (TUBB3) (red) that specifically stains enteric neurons and counterstaining with 4′,6-diamidino-2-phenylindole (DAPI) (blue), used to label cell nuclei. Scale bars = 12.5 μm. (**c**) Tridimensional growth of myenteric plexus cellular components after eight days of culture in neuronal medium, forming ganglion-like plexus structures (black arrows) (bright-field). Scale bars = 100 μm. (**d**) Representative immunofluorescent image of myenteric neurons after eight days of tridimensional culture in neuronal medium probed tubulin beta-III (TUBB3) (red) and DAPI (blue). Scale bars = 100 μm. (**e**) Small intestinal organoid after four days of culture in organoid media after passage (bright-field). Black arrows point to intestinal stem cells (ISCs) located at the bottom of the crypts, villi, and lumen of the organoid. Scale bars = 100 μm. (**f**) Representative immunofluorescent image of small intestinal organoids after four days of culture after passage in organoid medium stained with mucin-2 (MUC2) (green) labeling mucin secreted from goblet cells and DAPI (blue). Scale bars = 100 μm.

It takes approximately eight days of culture of the submucosal neurons to achieve ideal neural morphological features (Figure 4a and Figure S5a,b). The successful proliferation of submucosal enteric neurons is further confirmed through immunofluorescent staining, which utilizes the neuronal marker β-tubulin III (TUBB3) (Figure 4b).

As the myenteric plexus culture progresses to the eighth day, these cell clusters become interconnected through the extensions of neurites, closely resembling the natural arrangement observed in the myenteric plexus (Figure 4c and Figure S6a,b). The successful proliferation of enteric neurons is further confirmed through immunofluorescent staining (Figure 4d). This staining technique provides validation of the presence and development of enteric neurons within the cultured tissues.

After four days of culture of passed small intestinal organoids, they have proper morphology for immunocytochemical techniques (Figure 4e,f).

At this point, it becomes possible to initiate the co-culture of submucosal neurons, myenteric neurons, and small intestinal organoids. This co-culture system serves to create a complex cellular environment that accurately replicates the intricate interactions between the ENS and the intestinal epithelium.

To ensure optimal development and readiness for co-culture, a meticulous coordination of growth and culture timelines between the neuronal cultures and small intestinal organoids is crucial. The isolated enteric neurons are carefully positioned in close proximity to the small intestinal organoids, as this spatial arrangement plays a pivotal role in facilitating the establishment of neural connections between the neurons and the organoids. Finally, the co-culture system is cultured under controlled conditions that foster the growth of both the enteric neurons and the small intestinal organoids. By employing this strategic approach, a robust and physiologically relevant model system is established. This novel model enables investigations into the intricate interplay between the ENS and the intestinal epithelium in a controlled and reproducible manner.

During this process, digestion of the neuronal culture of the myenteric plexus is needed. The following steps outline the process of isolating neurons from the myenteric plexus and gathering neurons from the submucosal plexus:

1. Warm the 0.25% trypsin solution at 37 °C in a water bath.
2. Remove the current neuronal culture medium from each well of the 24-well plate using a pipette.
3. Add 500 μL of cold supplemented advanced DMEM/F12 media to each well and use a pipette with a bent tip to gently break up the gel and the crypts. Transfer the mixture to a 15 mL tube.
4. Spin the tube containing the crypts at 600 rpm at 4 °C.
5. Discard the supernatant.
6. Add 3 mL of warm 0.25% trypsin solution to the pellet.
7. Incubate the cells for digestion in a 37 °C water bath for 7 min.

Critical: Extended incubation with trypsin poses a risk of neuronal damage.

8. Neutralize the trypsin by adding 500 μL of cold FBS.
9. Centrifuge the cells for 8 min at 356× *g*.
10. While the cells are being centrifuged, place a sterilized 70 μm filter on top of a sterile 15 mL falcon tube.
11. Remove and discard the supernatant.
12. Gently resuspend the cell mixture by gently pipetting in 5 mL of DMEM/F12 media. Do not generate air bubbles.
13. Count the cell number using standard Trypan Blue staining and hemocytometry.
14. Calculate to seed at a density of 1.5 × 10^4^ enteric neurons from the myenteric plexus per 30 μL of Matrigel.
15. Calculate to seed at a density of 2.5 × 10^4^ enteric neurons from the submucosal plexus per 30 μL of Matrigel. Note the submucosal neurons are generally smaller in size compared to myenteric neurons due to functional specialization.
16. We are preparing 48 wells so therefore we collect the appropriate volume to gather 7.2 × 10^5^ enteric neurons from the myenteric plexus and 1.2 × 10^6^ isolated enteric neurons from the submucosal plexus.
17. Centrifuge the neuronal cells for 8 min at 356× *g*.
18. Remove and discard the supernatant.
19. Add 390 μL of ice-cold liquid Matrigel to 7.2 × 10^5^ isolated enteric neurons from the myenteric plexus.
20. Add 390 μL of ice-cold liquid Matrigel to 1.2 × 10^6^ isolated enteric neurons from the submucosal plexus.
21. Keep on ice.

Proceed by passing and breaking the small intestinal organoids as aforementioned.

1. Calculate to see crypts at a density of 150 crypts in 30 μL of liquid ice-cold Matrigel. We are plating 48 wells so 7200 crypts are diluted in 750 μL of ice-cold liquid Matrigel.
2. Keep on ice.

Proceed by carefully combining the isolated enteric neurons from the submucosa and from the myenteric plexus with the small intestinal organoids. If desired, continue culturing independently the cultures. These independent cultures can serve as independent controls.

1. Combine 390 μL of ice-cold liquid Matrigel containing 7.2 × 10^5^ isolated enteric neurons from the myenteric plexus, 390 μL of ice-cold liquid Matrigel containing 1.2 × 10^6^ isolated enteric neurons from the submucosal plexus, and 750 μL of ice-cold liquid Matrigel containing 7200 crypts.
2. With a pre-wetted pipette perform pipetting up and down motions for proper mixture.
3. Position droplets containing 30 μL of crypts embedded in Matrigel at the center of each pre-warmed well in the 24-well plate. Plate 48 wells. If immunofluorescence or immunohistochemical analysis is desired, plating should be performed on a 4-well chamber slide (Thermo Scientific, 154526).
4. Add 500 μL of complete small intestinal growth media to facilitate proper growth and innervations.
5. Transfer the plate to a CO_2_ incubator at 37 °C.
6. Allow the cells to grow for an additional four days.

By enabling the coexistence of enteric neurons and small intestinal organoids in a 3D culture environment, our technique allows for the investigation of various aspects of gut physiology and pathology. Researchers can explore the reciprocal interactions between neurons and the intestinal epithelium, including the influence of neuronal signaling on epithelial barrier function, nutrient absorption, motility responses, and antimicrobial responses. Additionally, this co-culture system can be utilized to study the pathogenesis of gastrointestinal disorders, such as irritable bowel syndrome (IBS), inflammatory bowel disease (IBD), and gastrointestinal motility disorders. In conclusion, our novel 3D in vitro co-culture technique offers a valuable platform for studying the intricate interplay between enteric neurons and the intestinal epithelium. It provides a more physiologically relevant model compared to traditional cell culture systems and holds significant potential for advancing our understanding of gut biology and developing targeted therapeutic strategies for gastrointestinal disorders.

**Figure 5:**
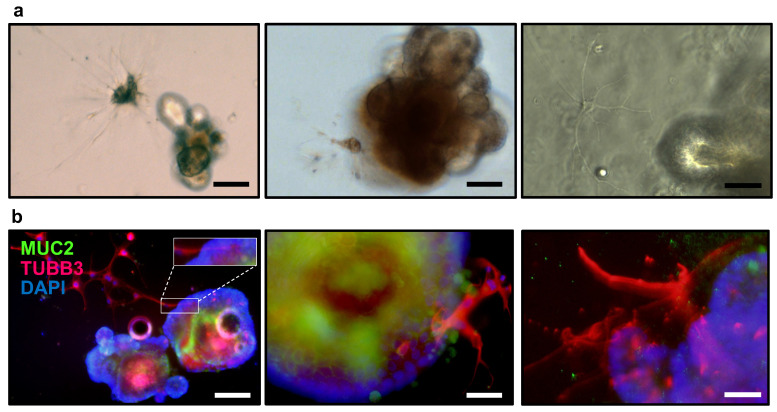
Co-culture of submucosal, myenteric, and small intestinal organoids grown in a three-dimensional culture for a period of four days. (**a**) Representative bright-field microscopy images of the co-culture grown in organoid medium after four days showcase neurons innervating or in close proximity to small intestinal organoids grown tridimensionally. The respective scale bars are 100 μm (white rectangles represent the 2× magnified area), 50 μm, and 25 μm. (**b**) A representative immunofluorescent image of the co-culture displays tubulin beta-III (TUBB3) in red, marking enteric neurons; mucin-2 (MUC2) in green, showing goblet cells; and DAPI in blue, marking cell nuclei. The scale bars for the image are 100 μm, 50 μm, and 25 μm, respectively.

After an additional four days of culture, specifically on the twelfth day, the enteric neurons innervate intestinal organoids and reach a stage where they exhibit desirable morphological characteristics (Figure 5a,b and Figure S7a–l).

### 3.2. Microinjection of Fluorescently Labeled Conjugates into Small Intestinal Organoids: A Versatile Technique for Investigating Cellular Integrity, Intestinal Permeability, and Neuronal-Epithelial Interactions

**Figure 6:**
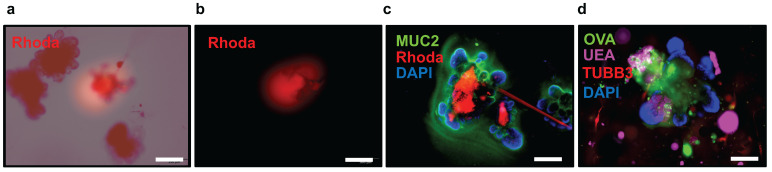
Nanoinjection of fluorescent-labeled conjugates to small intestinal organoids co-cultured with submucosal and myenteric neurons, grown for a period of four days. (**a**) A representative bright-field microscopy image depicts the nanoinjection process of a small intestinal organoid with fluorescently labeled dextran (rhodamine, red) while co-cultured with enteric neurons. The corresponding fluorescence capture is superposed on the image. Scale bar = 200 μm. (**b**) This image displays the superposed fluorescent capture as described in (**a**). Scale bar = 200 μm. (**c**) A representative immunofluorescent image showcases the co-culture following organoid nanoinjection with rhodamine-dextran (Rhoda, red), along with labeling for mucin-2 (MUC2) specifically targeting goblet cells. Additionally, DAPI staining in blue highlights the cell nuclei. A broken needle containing rhodamine-dextran nanoinjecting the organoid is visualized in the field. Scale bars = 200 μm. (**d**) Immunofluorescence performed on small intestinal organoids co-cultured with submucosal and myenteric neurons grown for four days and nanoinjected with fluorescein ovalbumin conjugate (OVA, green) into the organoids. Enteric neurons are labeled with tubulin beta-III (TUBB3) in red, goblet cells are stained with Ulex Europaeus Agglutinin (UEA) in magenta, and nuclei are labeled with DAPI in blue. Scale bars = 100 μm.

Furthermore, we have developed a method for microinjecting the luminal space of small intestinal organoids with fluorescently labeled dextran or fluorescently labeled ovalbumin (Figure 6a–d).

This technique possesses broad applicability, encompassing a diverse range of research areas. It can be used for assessing cellular integrity, intestinal permeability, and introducing specific bacterial species or pharmacological drugs relevant to digestive diseases. This method serves as a valuable tool for evaluating the intricate interplay between enteric neurons and IECs. The permeability of the intestinal epithelial barrier, which refers to how easily substances can pass through it, is an important factor in understanding diseases related to the rupture of intestinal homeostasis [16]. It is specifically related to the diffusion of small molecules through the paracellular space, which is the area between adjacent cells in the epithelium. IECs play crucial roles in absorbing water and nutrients, as well as establishing a protective barrier between the microbiota within the intestine and the host organism. This barrier is created by closely connected individual epithelial cells, which are tightly sealed together by proteins known as tight junctions. The permeability of this barrier, or how easily substances can pass through it, is controlled by the integrity of the cell membranes, tight junctions, and the processes of the epithelial cells involved in secretion and absorption [17,18,19]. Small molecules and electrolytes with a molecular weight of less than 300 Daltons can passively cross the tight junction barrier. Under normal conditions, the intestinal epithelium efficiently absorbs nutrients while preventing the movement of bacteria from the inside of the intestine into the body. However, under certain pathological conditions, the intestine can increase the passage of substances through the spaces between cells, affecting the permeability of the barrier [16,20,21,22]. This can lead to ineffective nutrient absorption and the translocation of bacteria and their products known as pathogen-associated molecular patterns (PAMPs) from the intestine into other organs. This increased permeability of the barrier can contribute to the development of chronic intestinal diseases, and it can also affect distant organs that filter and process the bacteria and associated PAMPs that have translocated from the intestine [16,17,20,21,22,23,24]. This co-culture protocol can be utilized to understand how the ENS affects permeability and regulates intestinal homeostasis in the presence or absence of different insults or treatments by injecting fluorescently labeled compounds.

**Figure 7:**
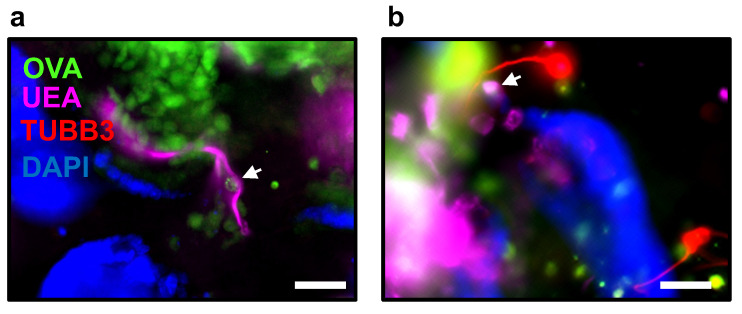
Nanoinjection of fluorescent-labeled conjugates allows for the visualization of goblet cell-associated antigen passages (GAPs). (**a**,**b**) Immunofluorescence performed on small intestinal organoids co-cultured with submucosal and myenteric neurons grown for four days and nanoinjected with fluorescein ovalbumin conjugate (OVA, green) into the organoids. Enteric neurons are labeled with tubulin beta-III (TUBB3) in red, goblet cells are stained with Ulex europaeus agglutinin (UEA) in magenta, and nuclei are labeled with DAPI in blue. Arrow points at a GAP. Scale bars = 25 μm.

Among other applications, the microinjection of small intestinal organoids with fluorescently labeled compounds could also be utilized to evaluate goblet cell-associated antigen passages (GAPs). In the small intestine, goblet cells regulate the intestinal immune response via GAPs formation. GAPs are ~5 μm in diameter and ~20 μm long and are formed after mucin secretion. GAPs deliver small soluble luminal antigens and bacteria to lamina propria dendritic cells (LP-DC) regulating adaptive immune responses [25]. GAP formation is dynamic, opening in response to ACh acting on the mAChR4 [26]. Therefore, the presence of ACh derived from the co-culture with submucosal and myenteric neurons would facilitate the formation of GAPs in small intestinal organoids. This can be observed as dextran-filled columns after microinjection with tetramethylrhodamine (TMR)-dextran 10,000 MW inside goblet cells or by detecting the presence of fluorescein-ovalbumin inside goblet cells (Figure 7a,b).

In order to microinject and subsequently perform immunofluorescence or immunohistochemical assessments, plating of co-cultured small intestinal organoids with submucosal and myenteric neurons should be performed on a 4-well chamber slide (Thermo Scientific, 154526).

Materials for Microinjection of Fluorescently Labeled Conjugates into Small Intestinal Organoids:

R-480 Nanoliter Microinjection Pump (RWD, #MM-500) (Supplementary Figure S8)

Mineral oil

3-Axis Motorized micromanipulator

MP-500 Micropipette puller

Microscope: Olympus Microscope IX71 Inverted with DIC and Fluorescence

2.5 mg/mL tetramethylrhodamine (TMR)-dextran 10,000 MW, lysine fixable (Life Technologies, D1817)

1 mg/mL ovalbumin, fluorescein conjugate (Life Technologies, O23020)

Protocol for Microinjection of Fluorescently Labeled Conjugates into Small Intestinal Organoids:

1. Prepare the microinjector according to the manufacturer’s instructions.
2. Position the microinjector near the microscope on a stable platform.
3. Use the micropipette puller to pull glass capillaries (TW100F-6, WPI, with a 1.0 mm outer diameter and 0.75 mm bore) under the following conditions: heat (400), filament (4), velocity (50), delay (250), pull (200). Aim for an inner diameter of approximately 15 μm at the tip of the capillary. Pull glass capillaries to a length of 5.5 cm. Ensure the glass capillary needles are sterilized.
4. Fill the glass capillary needle with mineral oil using a long steel needle.
5. Attach the capillary to the microinjector, ensuring that there is no air trapped inside by emptying the mineral oil from the capillary.
6. Prepare a small chamber and add fluorescently labeled dextran or ovalbumin to one of the wells. Fill the capillary with 50 μL of the fluorescently labeled compound, taking care to avoid any breakage of the capillary tip.
7. Move the co-culture of enteric neurons and the organoids chamber from the cell culture incubator to a microscope set up in close proximity to the microinjector.
8. Open the chamber lid and adjust the micromanipulator to position the capillary tip above the first organoid, just above the culture media. Use the *Z*-axis control of the micromanipulator to advance the capillary, first penetrating the Matrigel until reaching the organoid surface.
9. Carefully penetrate the organoid with the microcapillary tip and inject 50 nL of the fluorescently labeled compound.

Critical: Gentle penetration of the organoid is essential to prevent excessive epithelial damage.

10. Monitor the microinjection process by activating the fluorescence light source to excite the fluorophores and observe the emitted light from the fluorescently labeled compound within the organoid (Figure 6a,b).

Critical: To address concerns related to potential phototoxicity and cytoplasmic damage associated with fluorescence excitation in living tissue, take several precautions. Firstly, carefully optimize excitation parameters, such as intensity (20%) and short duration (1 min), to minimize the risk of phototoxicity. Secondly, it is advisable to include controls for phototoxicity effects. Thirdly, reduce the exposure time to a minimum (e.g., 160 ms) to further mitigate the potential for cellular damage. These measures collectively aim to strike a balance between obtaining high-quality fluorescence signals and ensuring the overall health and viability of the organoid during the microinjection process.

11. After injection, retract the needle to the media surface and proceed to the next organoid for microinjection.
12. Carry out the subsequent staining procedures.

## 4. Limitations

By recognizing and addressing the limitations of our co-culture system, we pave the way for improving the model. Firstly, during the 3D growth phase of the protocol, Matrigel, an animal-derived matrix product, introduces variability into the experiments, potentially impacting the growth characteristics of the organoids. To address this, exploring more defined matrix components and hydrogels could be valuable.

Microinjections, while useful for many applications, have their constraints, especially when assessing GAPs or studying the role of pathobionts or compounds that require introduction through the intestinal lumen. To overcome this, researchers could explore the use of monolayer cultures or inverted organoids, providing alternative methods to assess these specific aspects. Furthermore, incorporating reporter mice such as the B6.129-Gt(ROSA)26Sortm1(CAG-CHRM4*,-mCitrine)Ute/J model and advanced microscopy techniques such as two-photon or confocal microscopy could enhance the visualization. Furthermore, microinjection is a labor-intensive process, making it more suitable for simpler experiments, whereas for complex studies, working with monolayers or inverted organoids may be more advantageous.

Additionally, visualizing the submucosal plexus can be challenging, but promising strategies exist, such as employing neuronal markers or utilizing reporter mice targeting specific neuronal types. These approaches could help to visualize the submucosal plexus in the co-culture system, leading to a more comprehensive understanding of the complex cellular interactions within the intestinal environment.

Awareness regarding the ratio of organoids to myenteric and submucosal neuronal cells is crucial to obtaining numerous innervated organoids, ensuring representative results. Additionally, the highest magnification is recommended to study specific interactions of the ENS with different cell types and utilizing immunofluorescent techniques with specific cell-type markers is advised.

Inbred strains are genetically identical, and employing tissues from multiple animals could offer greater neuronal density for studying the neuronal cytoarchitecture along the intestine. We acknowledge the potential benefits of incorporating this alternative approach into our experimental design.

Unfortunately, neural activity assays were not conducted during the establishment of this protocol. However, there is an intention to expand and enhance this technique in the future.

By recognizing these challenges and incorporating advanced techniques, researchers can overcome the existing gaps in our methodology. Progressively improving this model will lead to a more robust and comprehensive platform for studying the relevant components within normal gastrointestinal physiology and during gastrointestinal diseases. These efforts will ultimately contribute to advancing our knowledge and improving the applicability of our co-culture system in exploring various aspects of intestinal biology.

## 5. Discussion

This innovative technique offers several advantages over previous approaches and opens new avenues of research. The utilization of a co-culture model provides a valuable opportunity to investigate intricate interplays between neurons and intestinal cells, thereby offering a more faithful representation of physiological conditions in comparison to conventional cell culture approaches. The co-culture of submucosal and myenteric neurons with intestinal organoids offers a model to gain deeper insights into the molecular pathways involved in gastrointestinal diseases. Additionally, the method presented here results in a mixed culture of enteric neurons and glia cells. The presence of glia is highly advantageous, as they contribute to the survival of the enteric neurons and the preservation of native receptor expression on neurons.

These advances hold promise in revolutionizing the treatment landscape for gastrointestinal diseases by facilitating the development of innovative therapies. Furthermore, the integration of myenteric neurons and submucosal neurons with organoids may uncover additional benefits that have yet to be fully realized. This technique could also shed light on the communication between neurons and the gut microbiota and/or the immune system if integrated into the co-culture, thus providing insights into the role of the microbiome in intestinal health and disease.

For instance, recognizing the significance of the microbiota’s influence on extraintestinal organs and associated conditions, such as the liver disease [23,27], the establishment of an integrated framework involving co-cultured intestinal organoids with the ENS, along with the introduction of specific pathobionts and isolated intestinal immune cells, could enable the examination of pathobiont roles in vitro. This approach would curtail the number of germ-free mice needed per model, reducing costs in studying the molecular mechanisms behind diseases. Moreover, it could facilitate drug screening and the exploration of therapeutic alternatives in a simulated in vitro setting. While this is just one instance, the concept holds potential for broader application to various gastrointestinal disorders or diseases linked to the gut-axis.

Additionally, this model may enable the study of neuropathies within the gastrointestinal system, offering new possibilities for therapy. Promising ENS stem cell therapies for enteric neuropathies are emerging. Initial trials should target diseases with specific neuron loss, like esophageal achalasia or possibly gastroparesis. Advances in refining cell isolation, improving culture methods, optimizing genetic engineering, and enhancing delivery strategies would lead to significant progress in the field [28]. While other techniques have been developed to investigate the ENS and the intestinal epithelium interface [29,30,31], the uniqueness and benefits of this particular approach lie in its complex representation of the cellular interactions with both enteric neural plexuses. Further studies focused on the proper identification of all isolated subtypes and their functional assays should be performed to evaluate the potential of this technology. In both rodents and humans, postnatal resident ENS neural stem/progenitor cells exist. This technology holds promise for discovering new treatments for enteric neuropathies with notable benefits such as the ability to generate enteric neurons educated to innervate and grow in the presence of IECs. Additionally, the utilization of patient-derived autologous cells may eliminate the need for immune suppression.

We have adhered to the guidelines outlined by Burns et al., which concentrate on establishing methods and approaches for identifying, isolating, purifying, expanding, and optimizing ENS stem cells and progenitors [28]. Utilizing cell sorting for specific neuronal subtypes would enhance purity, making it a valuable approach for studying particular neuronal subsets. To assess different cell types, it is recommended to test stem/progenitor molecular markers such as ret proto-oncogene (*Ret*), peptidyl arginine deiminase, type VI (*Padi6*), Nestin (*Nes*), neural cell adhesion molecule 1 (*Ncam1*), sex determining region Y (SRY)-box 2 (*Sox2*), SRY-box 10 (*Sox10*), antigen identified by monoclonal antibody Ki 67 (*Ki-67*), phospho-Histone 3, bromodeoxyuridine (BrDU), and 5-ethynyl-2′-deoxyuridine (EdU), using neuronal markers including tubulin beta 3 class III (*Tubb3*), ubiquitin carboxy-terminal hydrolase L1 (*Uchl1*), ELAV like RNA binding protein 3 (*Elavl3*), peripherin (*Prph*), achaete-scute family bHLH transcription factor 1 (*Ascl1*), microtubule-associated protein 2 (*Map2*), neurofilament, medium polypeptide (*Nefm*), 160/200-KDa neurofilament (NF), microtubule-associated protein tau (*Mapt*), *Vip*, nitric oxide synthase 1, neuronal (*Nos1*), choline acetyltransferase (*Chat*), acetylcholinesterase (*Ache*), calcitonin/calcitonin-related polypeptide, alpha (*Calca*), neuropeptide Y (*Npy*), peptide YY (*Pyy*), tachykinin 1 (*Tac1*), galanin and GMAP prepropeptide (*Gal*), tyrosine hydroxylase (*Th*), serotonin, dopamine beta-hydroxylase (*Dbh*), solute carrier family 1 (*Slc1a1*), and synaptophysin (*Syp*), as well as glial markers such as glial fibrillary acidic protein (*Gfap*), S100 protein, beta polypeptide, and neural (*S100b*). Although neural activity assays were not initially conducted, it is advisable to perform electrophysiological recordings. In addition, the validation of neurites containing relevant neurotransmitters should be performed. It is now evident that certain enteric neurons originate from Schwann cell precursors [32], and there is a likelihood that derived neurons exhibit distinct neurochemical phenotypes compared to others. Consequently, the presence of these neurons in our co-culture system is very likely, but it requires further investigation for confirmation.

Due to the complexity of this system, this technique offers a powerful tool to understand gastrointestinal diseases that have alterations in the ENS and the microbiome. By understanding the molecular mechanism behind these alterations, this model could facilitate the development of targeted therapies. Furthermore, it has promising implications in the field of regenerative medicine, offering a glimmer of hope to patients grappling with digestive diseases [33]. Taking into consideration the new advances in the field, this co-culture could be performed utilizing novel bioengineering designs, such as microfluidic devices, scaffold-based systems, and organoid-on-a-chip models [34,35,36], to ensure the reproducibility and functionality of our organoid culture systems. These cutting-edge techniques can replicate physiological fluid flow and create spatial arrangements of different cell types that could benefit the application. The protocol outlined in this manuscript is anticipated to generate valuable model systems for investigating a myriad of issues extending beyond the specific focal points highlighted in this manuscript.

In conclusion, the co-culture of myenteric neurons and submucosal neurons with organoids represents a groundbreaking advance in the field of organoid technology. The integration of ENS-organoids could play a pivotal role not only in helping to understand the intricate interplay between cells in the intestinal microenvironment but also holds promise for drug screening, gene editing, designing new therapies, modeling infectious diseases, and advancing regenerative medicine [33,37,38,39]. Its unique capabilities and potential for further discoveries make it a valuable tool for unraveling the complexity of gastrointestinal diseases and improving patient outcomes. There is a long path to successful clinical translation. Therefore, the improvement of the multicellular co-culture system, including material and technical enhancements, is essential to better simulate the in vivo intestinal environment and establish its efficacy. The development of this new system holds promise for future research and treatment of digestive diseases.

## Figures and Tables

**Table 1:** Krebs Solution.

Vf = 1 L	mg	Cf (mM)
Sodium chloride (NaCl) (Millipore Sigma, Burlington, MA, USA, S5886)	6895	118
Potasium chloride (KCl) (Millipore Sigma, P9333)	343	4.6
Sodium dihydrogen phosphate (NaH_2_PO_4_) (Millipore Sigma, 1.06370)	156	1.3
Magnesium sulfate (MgSO_4_) (Millipore Sigma, 746452)	144.5	1.2
Sodium bicarbonate (NaHCO_3_) (Millipore Sigma, S5761)	2100	25
Glucose (Millipore Sigma, D9434)	1980	11
Calcium chloride (CaCl_2_) (Millipore Sigma, C4901)	277.5	2.5
Gibco™ Antibiotic/Antimycotic, 100X (ThermoFisher, Waltham, MA, USA, 15240062), −20 °C	10 mL	1X

**Table 2:** Digestion Solution for Submucosal Plexus.

Vf = 10 mL		Cf (mM)
Collagenase, Type II, 1 g (ThermoFisher, 17101015)	13 mg	1.3 mg/mL
Bovine Serum Albumin Fraction V, heat shock from bovine serum, 250 g, (Roche, Basel, Switzerland, 3116956001), 4 °C	3 mg	0.3 mg/mL
Carbogen-bubbled Krebs solution	10 mL	

**Table 3:** Antibiotic cocktail (20X).

Vf = 10 mL	V_o_ (μL)	Cf
Ampicillin sodium solution 100 mg/mL (Goldbio, St. Louis, MO, USA, A-301-25), −20 °C	400	0.2 mg/mL
Gentamycin, 10 mg/mL (Millipore Sigma, G1272-10ML), 4 °C	1000	0.05 mg/mL
Gibco™ Antibiotic/Antimycotic, 100× (ThermoFisher, 15240062), −20 °C	2000	20X
Neomycin, 10 mg/mL (Millipore Sigma, N1142-20ML), 4 °C	1000	0.05 mg/mL
Metronidazole, 50 mg/mL (Millipore Sigma, M3761-5G), 4 °C	1000	0.25 mM
Vancomycin hydrochloride solution 200 mM (Millipore Sigma, 1709007-500MG), −20 °C	160	0.16 mM

**Table 4:** Neurobasal A media containing 0.2X antibiotics.

Vf = 500 mL	mL	Cf
Antibiotic cocktail 20X, −20 °C	5	0.2X
Neurobasal™-A Medium, minus phenol red (Gibco, 12349015), 4 °C	500	

**Table 5:** Glial-Derived Neurotrophic Factor (GDNF) solution.

Vf = 50 μL		Cf
Glial-Derived Neurotrophic Factor (GDNF) (Millipore Sigma, SRP3200), −80 °C	10 μg	10 μg/mL
ddH_2_O	1 mL	

**Table 6:** Enteric neuron media.

Vf = 50 mL	V_o_ (μL)	Cf
Gibco™ Sodium pyruvate, 100 mM (ThermoFisher, 11360070)	500	1 mM
Gibco™ GlutaMAX™ supplement, 100 mL, 100X (ThermoFisher, 35050061)	500	2
Gibco™ B-27™ supplement, 50X (ThermoFisher,17504044)	1000	1X
Glial-Derived Neurotrophic Factor (GDNF) solution, 10 μg/mL	50	0.01 μg/mL
Fetal Bovine Serum (FBS) heat inactivated (Omega Scientific, FB-02)	5000	10%
Antibiotic cocktail (20X)	2500	1X
Neurobasal A media containing 0.2X antibiotics	40,450	

**Table 7:** Small intestinal organoid culture materials.

Falcon 50 mL Conical Centrifuge Tubes (Corning, Corning, NY, USA, 1495949A)
Falcon 15 mL Conical Centrifuge Tubes (Fisher Scientific, 14-959-49B)
Biopioneer 50 mL Serological Pipet, 100/pack (Fisher Scientific, GEX500-S01)
Biopioneer 25 mL Serological Pipet 200/pack (Fisher Scientific, GEX250S01)
Biopioneer 10 mL Serological Pipet 200/pack (Fisher Scientific, GEX0100-S01)
Surgical forceps and scissors
Corning™ Disposable Vacuum Filter/Storage Systems 500 mL, 0.2 μm (Fisher Scientific, 430769)
Falcon^®^ 70 µm Cell Strainer, White, Sterile (Corning, 352350)
Whatman Puradisc 25 mm PES Syringe Filters, 0.2 µm, 50 pack, (50 units) (Whatman, Maidstone, UK, 6780-2502)
Cotton Tip Applicator 6″ 2′s Sterile 100/box (Dinarex, Montvale, NJ, USA, 4305)
Falcon^®^ 150 mm Tissue Culture -treated Cell Culture Dish with 20 mm Grid, 10/Pack, 100/Case, Sterile (Corning 353025)
Falcon^®^ 100 mm Tissue Culture-treated Cell Culture Dish, 20/Pack, 200/Case, Sterile (Corning, 353003)
Falcon^®^ 24-well Clear Flat Bottom Tissue Culture-treated Multiwell Cell Culture Plate, with Lid, Sterile, 50/Case, (Corning, 353047)
Syringe with BD Luer-Lok™ Tip, 30 mL (BD302832, VWR)
Syringe 27Gx1/2″ (BD#309623) 1 mL syringe, (Fisher Scientific, 1482687)
25G 5/8 Needle (BD# 305122), (Fisher Scientific, 14-826AA)
BD^®^ Needle 1/2 in. single use, sterile, 27 G (BD, 305109)
BD Precisionglide^®^ Needle 21G × 1 (0.8 mm × 25 mm), (BD, 305165)
Dissection Dish, Large, Black 3-Pack (Living systems instrumentations, DD-90-S-BLK-3PK)
Fisherbrand™ Razor Blades,100/pk, (Fisher Scientific, 12-640)

**Table 8:** Small intestinal organoid culture reagents.

Corning^®^ Matrigel^®^ Growth Factor Reduced (GFR) Basement Membrane Matrix, 10 mL (Millipore Sigma, CLS356231). Prepare 1 mL aliquots and store at −80 °C
Advanced DMEM/F12, 500 mL (ThermoFisher 12634010), 4 °C
Gibco™ GlutaMAX™ supplement (ThermoFisher, 35050061), 4 °C
Gibco™ (N-2-hydroxyethylpiperazine-N-2-ethane sulfonic acid) (HEPES) 1 M, 100 mL, (ThermoFisher, 15630080), 4 °C
Gibco™ Antibiotic/Antimycotic, 100X (ThermoFisher, 15240062), −20 °C
Gibco™ B-27™ supplement, 50X, 10 mL (ThermoFisher, 17504044). Prepare 400 μL aliquots and stored at −20 °C
Gibco™ N2 supplement, 100X, 5 mL (ThermoFisher, 17502048). Prepare 200 μL aliquots and store at −20 °C
Recombinant murine Noggin (mNoggin), 100 ug, (Peprotech, Cranbury, NJ, USA, 250-38). Prepare 100 μg/mL solution of mNoggin in 0.1% Bovine Serum Albumin (BSA) in Phosphate-Buffered Saline (PBS) to create a 1000X stock solution
1 mg of Gibco™ murine Epidermal Growth Factor (mEGF) Recombinant Protein (MG8043, fisherscientific). Prepare 500 μg/mL murine in 0.1% Bovine Serum Albumin (BSA) in Phosphate-buffered saline (PBS) to create a 10,000X stock solution. Store at −80 °C.
Fetal Bovine Serum (FBS) heat inactivated (Omega Scientific, Singapore, FB-02). Aliquot in 50 mL and store at −20 °C
N-Acetylcysteine, 5 G, (Millipore Sigma, A9165-5G), 4 °C. Prepare a 500 mM stock solution.
Gibco™ DPBS, no calcium, no magnesium (ThermoFisher, 14190136). Supplement with 5 mL of GibcoTM Antibiotic/Antimycotic, 100× (ThermoFisher, 15240062). 4 °C.
Ethylenediaminetetraacetic acid (EDTA) (0.5 M), pH 8.0, RNase-free (ThermoFisher, AM9260G), 4 °C
R-Spondin 1 conditioned media by a stable transfected 293T cell selected by zeocin expressing mouse R-spondin1 protein tagged with C-terminus HA and N-terminus Fc in the conditioned medium. (Kind gift from Calvin Kuo lab at Stanford University) (R&D Systems, Minneapolis, MN, USA, 3710-001-01).

**Table 9:** Preparation of 4 mL solution of 0.1% BSA in PBS 1X.

Vf = 4 mL		Cf
Bovine Serum Albumin Fraction V, heat shock from bovine serum, 250 g, (Roche, 3116956001), 4 °C	4 mg	0.1%
Phosphate Buffered Saline (PBS), 492 g, pH 7.4 (Fisher BioReagents, Pittsburgh, PA, USA, BP661-50). Prepare PBS 1X by mixing 9.84 g of PBS in 1 L of ddH_2_O and sterilize by autoclaving or filter sterilization, 4 °C.	4 mL	

**Table 10:** Preparation of 100 μg/mL solution of mNoggin in 0.1% BSA in PBS 1X to create a 1000× stock solution.

Vf = 1 mL		Cf
mNoggin, 100 μg, (PeproTech, 250-38), −80 °C	100 μg	100 μg/mL
0.1% Bovine Serum Albumin (BSA) in Phosphate-buffered saline (PBS), 4 °C	1 mL	

**Table 11:** Preparation of 500 μg/mL murine Epidermal Growth Factor (mEGF) Recombinant Protein in 0.1% BSA in PBS 1X to create a 10,000× stock solution.

Vf = 2 mL		Cf
1 mg of Gibco™ Epidermal Growth Factor (mEGF) Recombinant Protein (PMG8043, Fisher Scientific), −80 °C	1 mg	500 μg/mL
0.1% Bovine Serum Albumin (BSA) in Phosphate-Buffered Saline (PBS) 1X	2 mL	

**Table 12:** Preparation of 500 mM N-Acetylcysteine stock solution.

Vf = 5 mL		Cf
N-Acetylcysteine, 5 G, 163.19 g/mol, (Millipore Sigma, A9165-5G), 4 °C.	1 mg	500 mM
Sterile ddH_2_O.	5 mL	

**Table 13:** Preparation of supplemented advanced DMEM/F12 media.

Vf = 500 mL	V_o_ (mL)	Cf
Advanced DMEM/F12, 500 mL (ThermoFisher, 12634010), 4 °C	500	
Gibco™ GlutaMAX™ supplement, 100 mL, 100X (ThermoFisher, 35050061), 4 °C	5	1X
Gibco™ (N-2-hydroxyethylpiperazine-N-2-ethane sulfonic acid) (HEPES) 1 M, 100 mL, (ThermoFisher 15630080), 4 °C	5	10 mM
Gibco™ Antibiotic/Antimycotic, 100X (ThermoFisher, 15240062), −20 °C	5	1X

**Table 14:** Small intestinal organoid media.

Vf = 20 mL	V_o_	Cf
Gibco™ B-27™ supplement, 50X, 10 mL (ThermoFisher, 17504044), −20 °C	400 μL	1X
Gibco™ N2 supplement, 100X, 5 mL (ThermoFisher, 17502048), −20 °C	200 μL	1X
500 mM N-Acetylcysteine stock solution, −20 °C	50 μL	10 mM
R-Spondin 1 conditioned media, −20 °C	2000 μL	1X
500 μg/mL mEGF, 10,000X stock solution, −80 °C	2 μL	0.05 μg/mL
100 μg/mL mNoggin, 1000X stock solution, −80 °C	20 μL	0.1 μg/mL
Antibiotic cocktail (20X), −20 °C	1000 μL	1X
Supplemented advanced DMEM/F12, 4 °C	16,328 μL	

**Table 15:** Preparation of EDTA 2 mM in Dulbecco’s phosphate-buffered saline (DPBS) solution.

Vf = 25 mL	V_o_	Cf
EDTA (0.5 M), pH 8.0, RNase-free (ThermoFisher, AM9260G), 4 °C	100 μL	2 mM
Gibco™ DPBS, no calcium, no magnesium (ThermoFisher, 14190136) supplemented with 5 mL of GibcoTM Antibiotic/Antimycotic, 100× (ThermoFisher, 15240062). 4 °C.	24.9 mL	

**Table 16:** Prepare DPBS 1X solution with 10% FBS.

Vf = 100 mL	V_o_ (mL)	Cf
Fetal Bovine Serum (FBS) heat inactivated (Omega Scientific, FB-02), 4 °C	10	10%
Gibco™ DPBS, no calcium, no magnesium (ThermoFisher, 14190136) supplemented with 5 mL of GibcoTM Antibiotic/Antimycotic, 100× (ThermoFisher, 15240062), 4 °C	90	

## Data Availability

If reasonably requested or needed, data and samples/models will be made available for sharing to qualified parties provided that such a request does not compromise intellectual property interests, interfere with publication, or betray confidentiality. Data that are shared will include standards and notations required to accurately interpret the data, following commonly accepted practices in the field. Data and samples/materials will be available for access and sharing as soon as reasonably possible and no longer than two years after acquisition of the data.

## Supplementary Materials

The following supporting information can be downloaded at https://www.mdpi.com/article/10.3390/cells13100815/s1. Supplementary Figure S1. Isolated submucosal plexus, first day. Scale bars = 50 μm. Supplementary Figure S2. Isolated crypt, first day. Scale bars = 50 μm. Supplementary Figure S3. Isolated submucosal plexus, third day. Scale bars = 50 μm. Supplementary Figure S4. Isolated myenteric plexus, fourth day. (a) Neuronal projections forming a brush border. (b) Neuronal bodies. (c) Myenteric neurons and glia cells. Scale bars = 50 μm. Supplementary Figure S5. Isolated submucosal plexus, eighth day. Scale bars = 50 μm. Supplementary Figure S6. Isolated myenteric plexus, eighth day. Scale bars = 100, 50 μm, respectively. Supplementary Figure S7. Myenteric and submucosal neurons in co-culture with small intestinal organoids, after 4 days. Scale bars = 100 μm. Supplementary Figure S8. R-480 Nanoliter Microinjection Pump and 3-Axis motorized micromanipulator (#MM-500). Picture adapted from #MM-500 website.

## Abbreviations

3D, three-dimensional; *Ache*, acetylcholinesterase; *Ascl1*, achaete-scute family bHLH transcription factor 1; BMP, bone morphogenetic protein; BrDU, phospho-Histone 3, bromodeoxyuridine; BSA, bovine serum albumin; *Chat*, choline acetyltransferase; *Calca*, calcitonin/calcitonin-related polypeptide, alpha; DAPI, 4′,6-diamidino-2-phenylindole; Dbh, dopamine beta-hydroxylase; ddH2O, doubled-distilled water; DPBS, Dulbecco’s Phosphate-Buffered Saline; EDTA, Ethylenediaminetetraacetic acid; EdU, 5-ethynyl-2′-deoxyuridine; Elavl3, ELAV like RNA binding protein 3; EGF, Epidermal Growth Factor; ENS, enteric nervous system; Erk1/2, Extracellular Signal-Regulated Kinase 1 and 2; ESC, embryonic stem cells, FBS, Fetal Bovine Serum; *Gal*, galanin and GMAP prepropeptide; GAPs, goblet cell-associated antigen passages; GDNF, Glial-Derived Neurotrophic Factor; GFAP, glial fibrillary acidic protein; HA, hemagglutinin; HEPES, N-2-hydroxyethylpiperazine-N-2-ethane sulfonic acid; *Ki-67*, antigen identified by monoclonal antibody Ki 67; IBD, inflammatory bowel disease; IBS, irritable bowel syndrome; ICCs, interstitial cells of Cajal; IEC, intestinal epithelial cell; iPSC, induced pluripotent stem cell; ISC, intestinal stem cells; LGR5+, leucine-rich repeat-containing G protein-coupled receptor 5; LP-DC, lamina propria dendritic cells; mAChR4, muscarinic acetylcholine receptor 4; *Map2*, microtubule-associated protein 2; *Mapt*, microtubule-associated protein tau; MUC2, mucin-2; *Ncam1*, neural cell adhesion molecule 1; *Nefm*, neurofilament, medium polypeptide; *Nes*, Nestin; NF, 160/200-KDa neurofilament; *Nos1*, nitric oxide synthase 1, neuronal; *Npy*, neuropeptide Y; *Padi6*, peptidyl arginine deiminase, type VI (*Padi6*); PAMPs, pathogen-associated molecular patterns; PBS, phosphate-buffered saline; *Prph*, peripherin; *Pyy*, peptide YY; *Ret*, ret proto-oncogene (*Ret*); *Slc1a1*, solute carrier family 1; *S100b*, S100 protein, beta polypeptide, neural; *Sox2*, sex determining region Y (SRY)-box 2; *Sox10*, SRY-box 10; *Syp*, synaptophysin; *Tac1*, tachykinin 1; TGR5, G-protein coupled bile acid receptor 1; TMR, tetramethylrhodamine-dextran; Th, tyrosine hydroxylase; TUBB3, neuron marker β-tubulin III; *Tubb3*, tubulin beta 3 class III; *Uchl1*, ubiquitin carboxy-terminal hydrolase L1; UEA-I, Ulex Europaeus Agglutinin I; VIP, vasoactive intestinal peptide; VIPR2, VIP receptor type 2.

## References

[1] Almeqdadi M., Mana M.D., Roper J., Yilmaz O.H. (2019). Gut organoids: Mini-tissues in culture to study intestinal physiology and disease. Am. J. Physiol. Cell Physiol..

[2] Amen A.M., Ruiz-Garzon C.R., Shi J., Subramanian M., Pham D.L., Meffert M.K. (2017). A Rapid Induction Mechanism for Lin28a in Trophic Responses. Mol. Cell.

[3] Lin L.F., Doherty D.H., Lile J.D., Bektesh S., Collins F. (1993). GDNF: A glial cell line-derived neurotrophic factor for midbrain dopaminergic neurons. Science.

[4] Fairman W.A., Vandenberg R.J., Arriza J.L., Kavanaugh M.P., Amara S.G. (1995). An excitatory amino-acid transporter with properties of a ligand-gated chloride channel. Nature.

[5] Newsholme P., Lima M.M., Procopio J., Pithon-Curi T.C., Doi S.Q., Bazotte R.B., Curi R. (2003). Glutamine and glutamate as vital metabolites. Braz. J. Med. Biol. Res..

[6] Haramis A.P., Begthel H., van den Born M., van Es J., Jonkheer S., Offerhaus G.J., Clevers H. (2004). De novo crypt formation and juvenile polyposis on BMP inhibition in mouse intestine. Science.

[7] Date S., Sato T. (2015). Mini-gut organoids: Reconstitution of the stem cell niche. Annu. Rev. Cell Dev. Biol..

[8] Frey M.R., Golovin A., Polk D.B. (2004). Epidermal growth factor-stimulated intestinal epithelial cell migration requires Src family kinase-dependent p38 MAPK signaling. J. Biol. Chem..

[9] Suzuki A., Sekiya S., Gunshima E., Fujii S., Taniguchi H. (2010). EGF signaling activates proliferation and blocks apoptosis of mouse and human intestinal stem/progenitor cells in long-term monolayer cell culture. Lab. Investig..

[10] de Lau W., Peng W.C., Gros P., Clevers H. (2014). The R-spondin/Lgr5/Rnf43 module: Regulator of Wnt signal strength. Genes Dev..

[11] Hamnett R., Dershowitz L.B., Sampathkumar V., Wang Z., Gomez-Frittelli J., De Andrade V., Kasthuri N., Druckmann S., Kaltschmidt J.A. (2022). Regional cytoarchitecture of the adult and developing mouse enteric nervous system. Curr. Biol..

[12] Sato T., Vries R.G., Snippert H.J., van de Wetering M., Barker N., Stange D.E., van Es J.H., Abo A., Kujala P., Peters P.J. (2009). Single Lgr5 stem cells build crypt-villus structures in vitro without a mesenchymal niche. Nature.

[13] Bixby S., Kruger G.M., Mosher J.T., Joseph N.M., Morrison S.J. (2002). Cell-intrinsic differences between stem cells from different regions of the peripheral nervous system regulate the generation of neural diversity. Neuron.

[14] Zakhem E., Rego S.L., Raghavan S., Bitar K.N. (2015). The appendix as a viable source of neural progenitor cells to functionally innervate bioengineered gastrointestinal smooth muscle tissues. Stem Cells Transl. Med..

[15] Kriegstein A., Alvarez-Buylla A. (2009). The glial nature of embryonic and adult neural stem cells. Annu. Rev. Neurosci..

[16] Wang L., Llorente C., Hartmann P., Yang A.M., Chen P., Schnabl B. (2015). Methods to determine intestinal permeability and bacterial translocation during liver disease. J. Immunol. Methods.

[17] Sun Z., Wang X., Andersson R. (1998). Role of intestinal permeability in monitoring mucosal barrier function. History, methodology, and significance of pathophysiology. Dig. Surg..

[18] Duffey M.E., Hainau B., Ho S., Bentzel C.J. (1981). Regulation of epithelial tight junction permeability by cyclic AMP. Nature.

[19] Simon D.B., Lu Y., Choate K.A., Velazquez H., Al-Sabban E., Praga M., Casari G., Bettinelli A., Colussi G., Rodriguez-Soriano J. (1999). Paracellin-1, a renal tight junction protein required for paracellular Mg^2+^ resorption. Science.

[20] Hollander D. (1988). Crohn’s disease—A permeability disorder of the tight junction?. Gut.

[21] Katz K.D., Hollander D., Vadheim C.M., McElree C., Delahunty T., Dadufalza V.D., Krugliak P., Rotter J.I. (1989). Intestinal permeability in patients with Crohn’s disease and their healthy relatives. Gastroenterology.

[22] Wyatt J., Vogelsang H., Hubl W., Waldhoer T., Lochs H. (1993). Intestinal permeability and the prediction of relapse in Crohn’s disease. Lancet.

[23] Llorente C., Schnabl B. (2015). The gut microbiota and liver disease. Cell Mol. Gastroenterol. Hepatol..

[24] Anand S., Mande S.S. (2022). Host-microbiome interactions: Gut-Liver axis and its connection with other organs. NPJ Biofilms Microbiomes.

[25] McDole J.R., Wheeler L.W., McDonald K.G., Wang B., Konjufca V., Knoop K.A., Newberry R.D., Miller M.J. (2012). Goblet cells deliver luminal antigen to CD103+ dendritic cells in the small intestine. Nature.

[26] Knoop K.A., McDonald K.G., McCrate S., McDole J.R., Newberry R.D. (2015). Microbial sensing by goblet cells controls immune surveillance of luminal antigens in the colon. Mucosal Immunol..

[27] Bruellman R., Llorente C. (2021). A Perspective Of Intestinal Immune-Microbiome Interactions In Alcohol-Associated Liver Disease. Int. J. Biol. Sci..

[28] Burns A.J., Goldstein A.M., Newgreen D.F., Stamp L., Schafer K.H., Metzger M., Hotta R., Young H.M., Andrews P.W., Thapar N. (2016). White paper on guidelines concerning enteric nervous system stem cell therapy for enteric neuropathies. Dev. Biol..

[29] Workman M.J., Mahe M.M., Trisno S., Poling H.M., Watson C.L., Sundaram N., Chang C.F., Schiesser J., Aubert P., Stanley E.G. (2017). Engineered human pluripotent-stem-cell-derived intestinal tissues with a functional enteric nervous system. Nat. Med..

[30] Levin D.E., Mandal A., Fleming M.A., Bae K.H., Gerry B., Moore S.R. (2021). Intestinal crypt-derived enteroid coculture in presence of peristaltic longitudinal muscle myenteric plexus. Biol. Methods Protoc..

[31] Fattahi F., Steinbeck J.A., Kriks S., Tchieu J., Zimmer B., Kishinevsky S., Zeltner N., Mica Y., El-Nachef W., Zhao H. (2016). Deriving human ENS lineages for cell therapy and drug discovery in Hirschsprung disease. Nature.

[32] Espinosa-Medina I., Jevans B., Boismoreau F., Chettouh Z., Enomoto H., Muller T., Birchmeier C., Burns A.J., Brunet J.F. (2017). Dual origin of enteric neurons in vagal Schwann cell precursors and the sympathetic neural crest. Proc. Natl. Acad. Sci. USA.

[33] Okamoto R., Shimizu H., Suzuki K., Kawamoto A., Takahashi J., Kawai M., Nagata S., Hiraguri Y., Takeoka S., Sugihara H.Y. (2020). Organoid-based regenerative medicine for inflammatory bowel disease. Regen. Ther..

[34] Ingber D.E. (2022). Human organs-on-chips for disease modelling, drug development and personalized medicine. Nat. Rev. Genet..

[35] Gjorevski N., Sachs N., Manfrin A., Giger S., Bragina M.E., Ordonez-Moran P., Clevers H., Lutolf M.P. (2016). Designer matrices for intestinal stem cell and organoid culture. Nature.

[36] Shin W., Kim H.J. (2022). 3D in vitro morphogenesis of human intestinal epithelium in a gut-on-a-chip or a hybrid chip with a cell culture insert. Nat. Protoc..

[37] Roper J., Tammela T., Akkad A., Almeqdadi M., Santos S.B., Jacks T., Yilmaz O.H. (2018). Colonoscopy-based colorectal cancer modeling in mice with CRISPR-Cas9 genome editing and organoid transplantation. Nat. Protoc..

[38] Watanabe S., Kobayashi S., Ogasawara N., Okamoto R., Nakamura T., Watanabe M., Jensen K.B., Yui S. (2022). Transplantation of intestinal organoids into a mouse model of colitis. Nat. Protoc..

[39] Fumagalli A., Suijkerbuijk S.J.E., Begthel H., Beerling E., Oost K.C., Snippert H.J., van Rheenen J., Drost J. (2018). A surgical orthotopic organoid transplantation approach in mice to visualize and study colorectal cancer progression. Nat. Protoc..

