# Supplementary material for: Isolation of Myenteric and Submucosal Plexus from Mouse Gastrointestinal Tract and Subsequent Co-Culture with Small Intestinal Organoids
Source: Cells. 2024 May 10;13(10):815. doi: 10.3390/cells13100815 (PMC11120043; doi:10.3390/cells13100815)
Supplement: Supplementary file 1 [file cells-13-00815-s001.zip › Cells_Revised_Supplementary_Figures.pdf]

Supplementary information

a

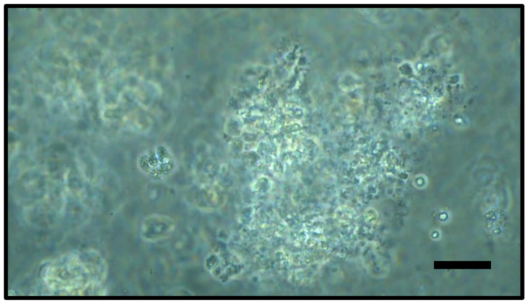

Figure S1. Isolated submucosal plexus, first day. Scale bars = 50 µm

a

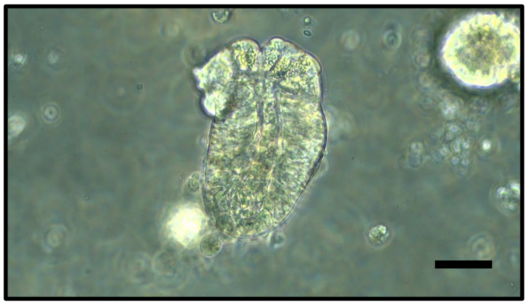

Figure S2. Isolated crypt, first day. Scale bars = 50 µm

a

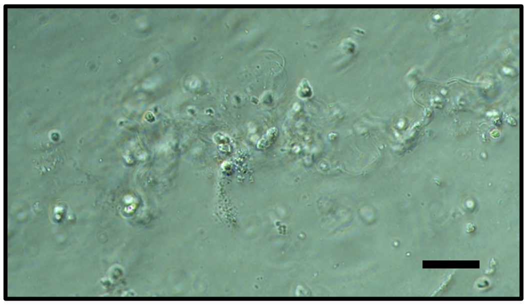

Figure S3. Isolated submucosal plexus, third day. Scale bars = 50 µm

a b c

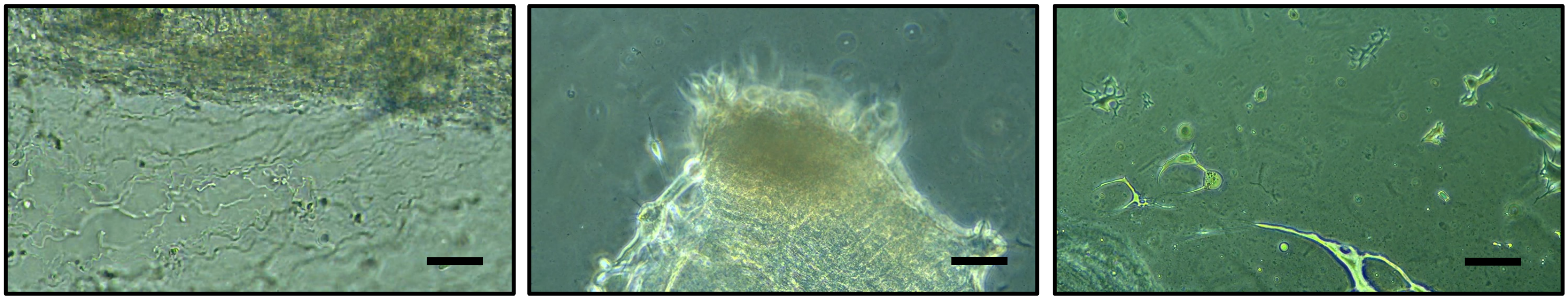

Figure S4. Isolated myenteric plexus, fourth day. (a) Neuronal projections forming a brush border. (b) Neuronal bodies. (c) Myenteric neurons and glia cells. Scale bars = 50 µm

a b

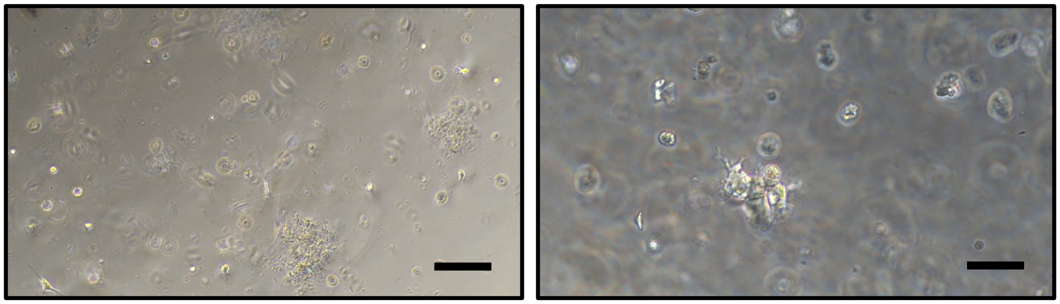

Figure S5. Isolated submucosal plexus, eighth day. Scale bars = 50 µm

a b

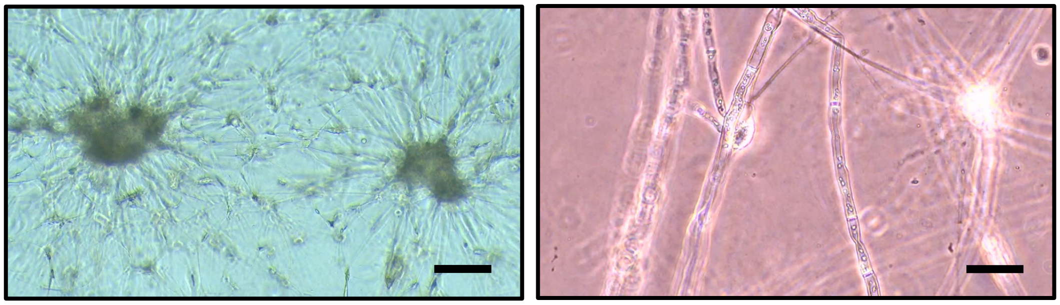

Figure S6. Isolated myenteric plexus, eighth day. Scale bars = 100, 50 µm respectively.

Supplementary information

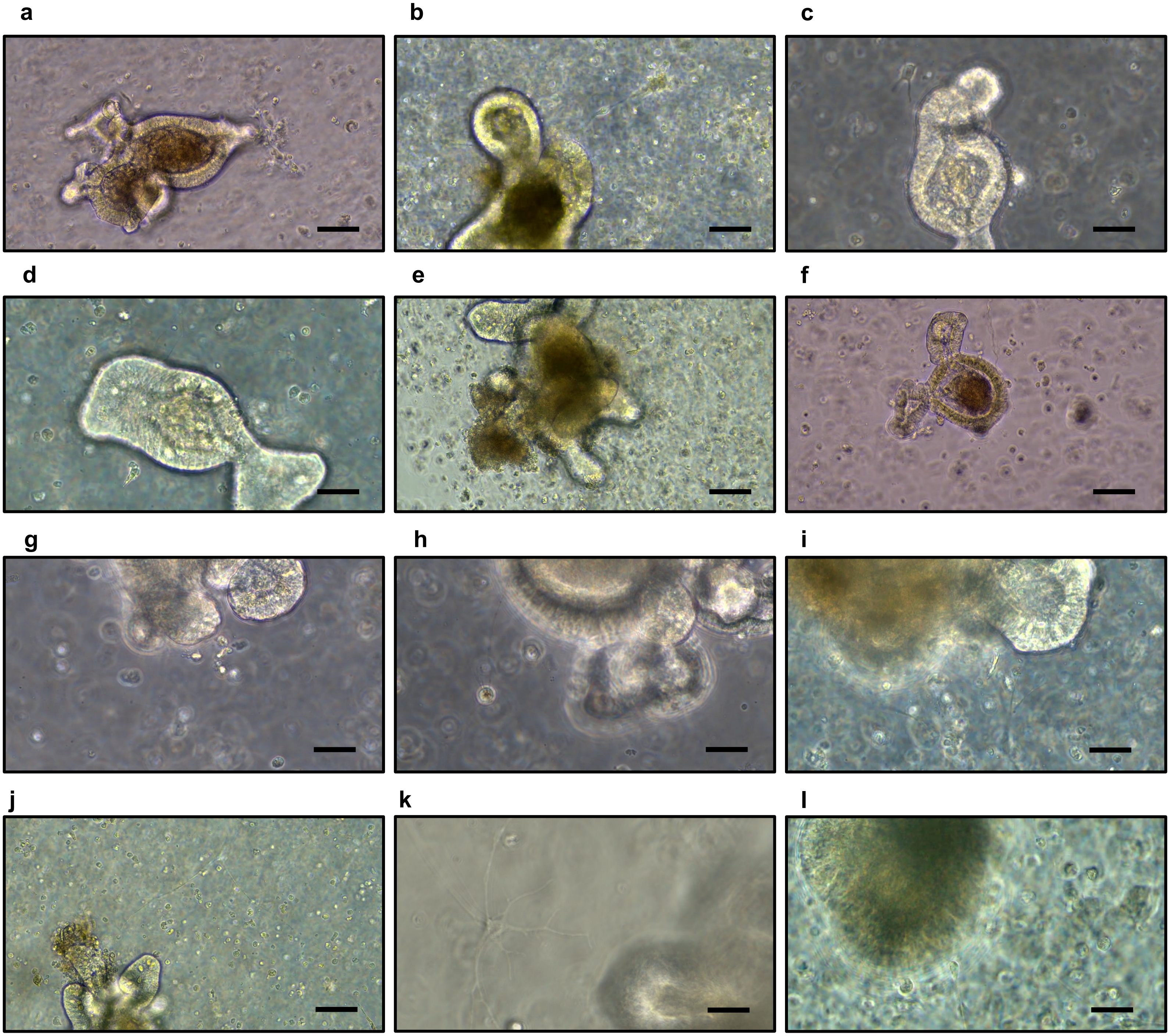

Figure S7. Myenteric and submucosal neurons in co-culture with small intestinal organoids, after 4 days. Scale bars = 100 µm.

Supplementary information

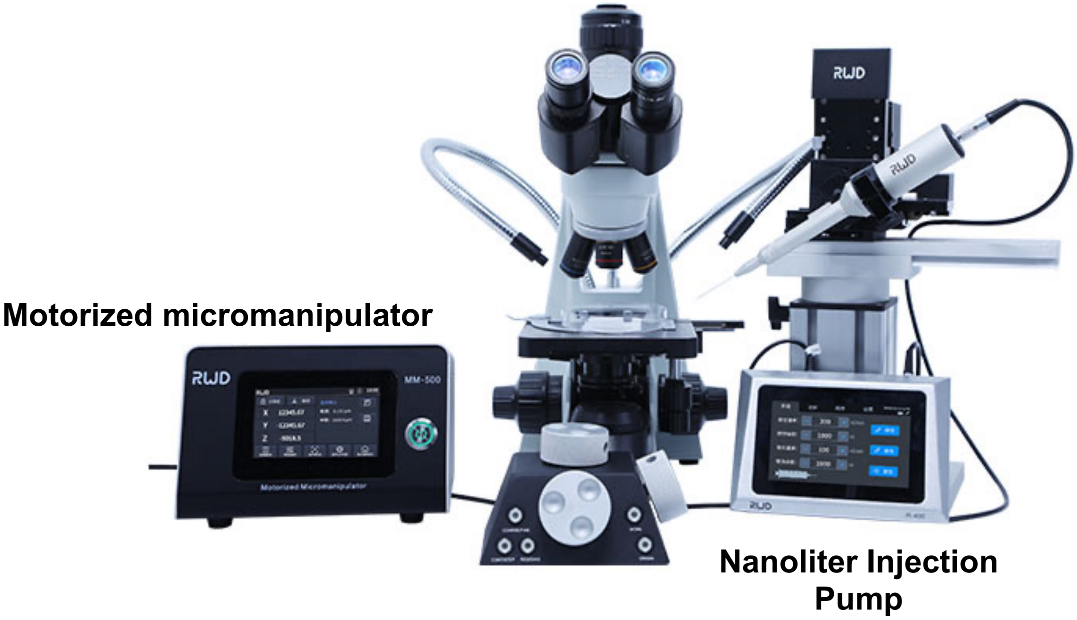

Figure S8. R-480 Nanoliter Microinjection Pump and 3-Axis motorized micromanipulator (#MM-500). Picture adapted from www.rwdstco.com website.
